# Characterization and Application of PVDF and Its Copolymer Films Prepared by Spin-Coating and Langmuir–Blodgett Method

**DOI:** 10.3390/polym11122033

**Published:** 2019-12-08

**Authors:** Zerun Yin, Bobo Tian, Qiuxiang Zhu, Chungang Duan

**Affiliations:** Key Laboratory of Polar Materials and Devices (MOE), Department of Electronics, East China Normal University, Shanghai 200241, China; yinzerun@foxmail.com (Z.Y.); cgduan@clpm.ecnu.edu.cn (C.D.)

**Keywords:** poly(vinylidene fluoride), organic ferroelectric polymers, memory, synapse

## Abstract

Poly(vinylidene fluoride) (PVDF) and its copolymers are key polymers, displaying properties such as flexibility and electroactive responses, including piezoelectricity, pyroelectricity, and ferroelectricity. In the past several years, they have been applied in numerous applications, such as memory, transducers, actuators, and energy harvesting and have shown thriving prospects in the ongoing research and commercialization process. The crystalline polymorphs of PVDF can present nonpolar α, ε phase and polar β, γ, and δ phases with different processing methods. The copolymers, such as poly(vinylidene fluoride-trifluoroethylene) (P(VDF-TrFE)), can crystallize directly into a phase analogous to the β phase of PVDF. Since the β phase shows the highest dipole moment among polar phases, many reproducible and efficient methods producing β-phase PVDF and its copolymer have been proposed. In this review, PVDF and its copolymer films prepared by spin-coating and Langmuir–Blodgett (LB) method are introduced, and relevant characterization techniques are highlighted. Finally, the development of memory, artificial synapses, and medical applications based on PVDF and its copolymers is elaborated.

## 1. Introduction

Ferroelectric thin films have attracted increasing interest because of their unique features, which can be exploited in many technologies and fields, such as nonvolatile memories [1,2], sensors, actuators, and energy harvesting. Compared with bulk ceramics, ferroelectric thin films show great advantages for specified applications, including energy efficiency and scalability [3]. As important members of ferroelectrics, organic ferroelectric polymers display flexibility, dielectric properties, electroactive response, and are produced via a simple fabrication process, which could allow their use for flexible displays and photovoltaic cells [4,5]. As organic ferroelectric materials, poly(vinylidene fluoride) (PVDF) and its copolymers typically show better overall performance than their counterparts (e.g., nylon-11 [6], croconic acid [7], and diisopropylammonium bromide (DIPA-B) [8]), thus they have been tested and adopted in a growing number of potential applications [9].

**Figure 1:**
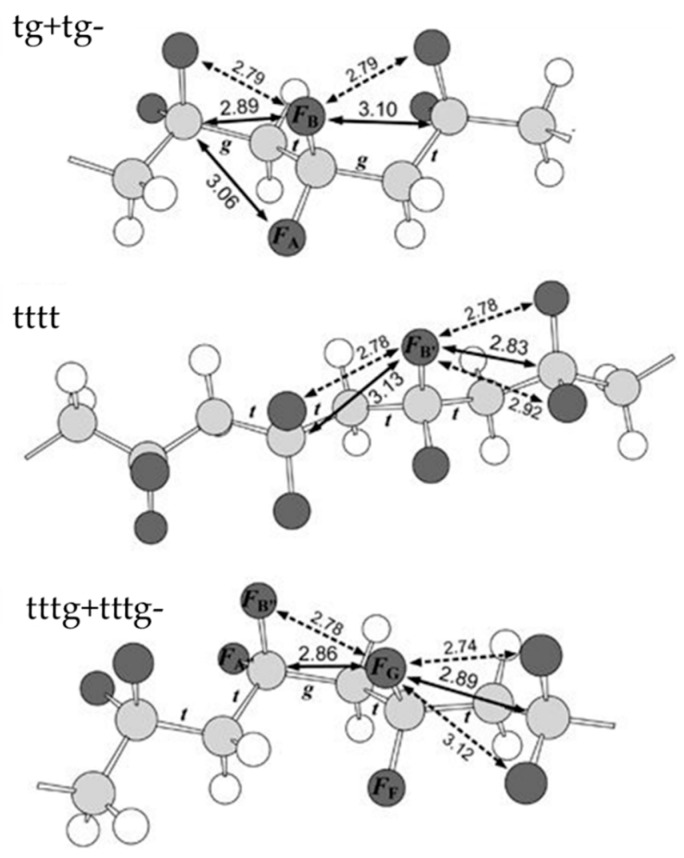
Three representative chain conformations of tg^+^tg^–^ for the α and δ phase, tttt for the β phase, and tttg^+^tttg^–^for γ and ε phase. Reproduced from [12], with permission from Springer Nature, 2012.

PVDF, a nonconjugated linear fluorinated hydrocarbon, is formed by the repeating unit –(CH_2_–CF_2_)–, which carries a vacuum dipole moment of *μ_ν_* = 7 × l0^−30^ Cm (2 Debyes) pointing roughly from the negative fluorine atom to the positive hydrogen atom [10,11]. PVDF is reported to be able to crystallize into at least five polymorphs, identified as α, β, γ, δ, and ε phases on the basis of the different chain conformations and packing of molecules [4,11,12]. With trans bond (*t*) for a dihedral angle of about 180° and left/right gauche bond (g^+^/g^–^) for dihedral angles of about ±60°, β phase shows all-trans (tttt) chain conformation; α and δ phase are tg^+^tg^−^, while γ and ε phases are tttg^+^tttg^−^ [13]. Figure 1 shows the three chain conformations optimized by density functional theory (DFT) calculation [12]. The chain conformations and packing of the molecules directly affect the orientation of dipoles which are rigidly attached to the chain [10]. Thus, among these polymorphs, the α phase shows no macroscopic polarization, while the remaining β, γ, and δ phases are polar. Roughly, by summing up the aforementioned *μ*_ν_ in a unit volume, the spontaneous polarization of the β phase is calculated to be 0.13 C/m^2^, about twice that of γ and δ phases [10]. More detailed information about the dipole moment and the polarizability has also been derived from the DFT calculations [14,15,16] and the molecular dynamics simulations (MDS) [17].

**Figure 2:**
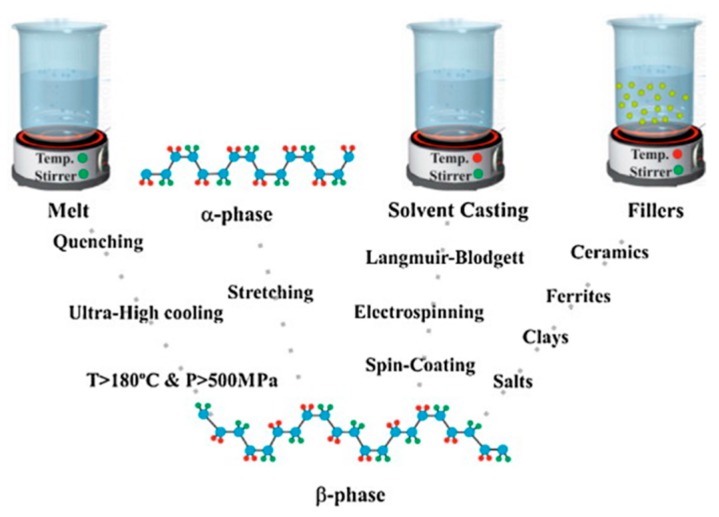
Approaches for obtaining the β phase of PVDF. From the melt, the α phase, or by solvent casting and adding fillers. Reproduced form [18], with permission from Elsevier, 2014.

Higher spontaneous polarization of β phase makes it present better pyroelectric, piezoelectric, and ferroelectric properties, when compared to the other four phases and some of PVDF’s counterparts. There is a brief comparison listed in Table 1. Many approaches to obtaining β phase have been suggested, as shown in Figure 2. The melt of PVDF needs to be subjected to specific conditions (e.g., ultra-fast cooling, and high pressure and temperature) to form β phase. Mechanical stretching is also commonly used to convert the α phase of PVDF to β phase. Solvent casting and the addition of fillers provide more convenient processes [18]. Additionally, copolymers of PVDF such as poly(vinylidene fluoride-trifluoroethylene) (P(VDF-TrFE)) and poly(vinylidene fluoride-tetrafluoroethylene) (P(VDF-TeFE)), have offered a straightforward approach to obtaining the electroactive phase [10]. Amid various methods proposed to obtain the β phase of PVDF and its copolymer, both spin-coating and Langmuir–Blodgett (LB) methods have proven to be effective [18,19].

In this review, the preparation of PVDF and its copolymer films via spin-coating method and Langmuir–Blodgett method are introduced. Based on these films, the mechanism of polarization switching is then summarized. Finally, several promising applications using the aforementioned films (e.g., electronic and medical devices) are outlined.

## 2. Preparation

### 2.1. Spin-Coating Method

Due to the simple process and good quality of prepared films, there has been a considerable amount of research on prompting the formation of β phase in spin-coated PVDF films [18,22,25,35,36,37,38,39,40,41,42,43,44,45]. The general process can roughly be divided into three steps: solution preparation, spin-coating, and annealing.

**Figure 3:**
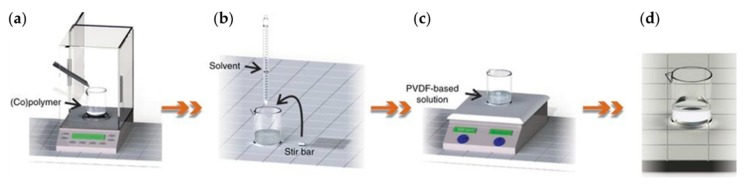
Procedure of preparing PVDF-based solution: (**a**) weigh the solute; (**b**) add solvent and magnetic stir bar; (**c**) magnetic stirring; (**d**) a transparent and homogeneous solution is obtained. Adapted from [19], with permission from Springer Nature, 2018.

Figure 3 shows the general process flow of preparing a PVDF-based solution for spin-coating. In the process flow, it is notable that the solvent used, nucleating fillers added, and concentration influence the subsequent steps. Firstly, as PVDF and its copolymers are dissolvable in many kinds of organic polar solvents (e.g., diethyl carbonate, dimethylformamide (DMF), N-methyl-2-pyrrolidone (NMP), and dimethylsulfoxide (DMSO)), subtle variations deriving from the solvent used have been reported. Nishiyama et al. evaluated how the solvent evaporation rate and solvent type influence the crystal formation in spin-coated PVDF films over time, and concluded that both the polymer–solvent electrostatic interactions and the evaporation conditions play important roles [42]. Additionally, by adding nucleating fillers like clay [46], hydrated salt [35], or nanoparticles such as CeO_2_ [47], palladium [48], or gold [49], the electroactive β phase can be enhanced. The nucleation mechanism varies for specific fillers; therefore, important theories such as transcrystallization behavior [47,50] have been set up to describe the main interactions leading to nucleation. In most studies about spin-coating PVDF and its copolymers, the solution concentration is a factor that partially determines the thickness of the resulting film, and the rotational speed is another [25,40,41,43]. Spin-coating generally produces thicker films (>60 nm), but it is reported that films thinner than 10 nm can be achieved with improved experimental conditions [51,52,53].

In the second step, the substrate temperature, rotational speed, and humidity are the main conditions that need to be adjusted to promote the formation of β phase and reduce the surface roughness of thin films. Ramasundaram et al. prepared a PVDF thin film containing crystalline β phase by heat-controlled spin-coating directly, without adding fillers or making post-treatments [38]. Samples were prepared under different temperatures, and the results show that elevated temperatures (40, 50, 60, and 70 °C) facilitated the crystallization of β phase, while low temperatures (20 and 30 °C) promoted α phase crystallization [38]. The influence of rotation speed on the β-phase content in PVDF films was studied, and the conclusion showed that high rotation speed and low solvent concentration benefited the formation of β phase in the films [25,40]. This can be ascribed to the shear and elongation forces stretching the polymer chains, promoting the α-to-β-phase transformation during rotation [40].

Finally, annealing the film in an elevated temperature is commonly done to improve the morphology and β-phase content of films. Cardoso et al. point out that β-phase content decreases with elevated annealing temperature, because the faster solvent evaporation and film crystallization facilitate the formation of α phase [40]. However, without annealing, the solvent evaporation rate is so slow that pores form in the resulting films, which may hamper their subsequent application [39]. Annealing between 30 and 80 °C immediately after spin-coating increases the solvent evaporation rate and ensures a film with both smooth and flat surface and high β-phase content.

Aside from annealing, there are other post-treatments, such as stretching and electrical polarizing, that can be successively applied to the films to improve β phase or generate other phases [19]. Note that the overlooked polar δ-phase PVDF can be formed by applying a short electrical pulse to PVDF film spin-coated under elevated substrate temperatures [51]. Kim et al. investigated the effect of alkaline treatment on spin-coated P(VDF-TrFE) films, and they suggested that the treated films showed degradation of the remanent polarization and small change in the surface roughness [54].

Compared with PVDF, copolymers of PVDF with trifluoroethylene (TrFE) and tetrafluoroethylene (TeFE) crystallize readily from the melt into polar β phase, and the cause was well analyzed by Furukawa et al. [10]. After proper post-treatment, the crystallinity of the copolymers can exceed 90% [13]. Thus the abovementioned spin-coating method is also applicable for P(VDF-TrFE) and P(VDF-TeFE), and the process can be even more convenient than PVDF [25].

### 2.2. Langmuir–Blodgett Deposition

By spin-coating, it is convenient to prepare β-phase PVDF and P(VDF-TrFE) films with thickness ranging from tens to thousands of nanometers and even thicker. However, to make ultra-thin or even two-dimensional films at a thickness of several nanometers, LB deposition is superior [27,55,56].

**Figure 4:**
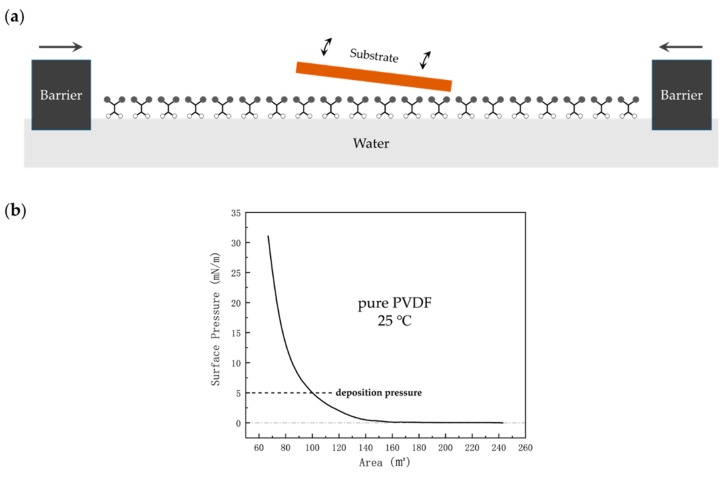
(**a**) Diagram of transferring monolayers to a solid substrate in the LB method; (**b**) measured isotherm of the surface pressure versus the remaining trough area while preparing pure PVDF LB films.

Together with Blodgett and Schaefer, Langmuir developed technology for obtaining single/multiple molecular monolayer(s) by spreading the amphiphilic molecules onto water and then transferring to a substrate vertically or horizontally [11], as shown in Figure 4a. Although PVDF and its copolymers are not amphiphiles, amphiphilic properties are not necessary for LB deposition [57]. The Langmuir films of PVDF and its copolymer can be prepared from solutions of 0.01%–0.10% weight concentration in DMSO or acetone. By spreading a small amount of solution, the molecules floating on the water surface can be compressed by a moving barrier to form a dense monolayer. Important information about the monolayer state on a water surface can be obtained from the pressure–area isotherm—the dependence of the surface pressure on film area, as illustrated in Figure 4b.

Since both acetone and DMSO are soluble in water, they can carry a significant quantity of the material into the bulk of the water. In order to minimize the loss of material, precautions such as depositing the solution in small drops and allowing time between drops for the solution to spread out on the surface must be taken [11]. It is proposed that when the surface pressure reaches about 5 mN/m, polymer molecules are closely packed with the polymer chains on the surface plane of water [27,57]. By depositing at a surface pressure slightly above 5 mN/m, films of 2–3 nm thickness, with several modular layers, can be obtained, and the defects in the film are more obvious, as well [41]. Depositing at significantly high surface pressures forms poor samples [11].

To get thicker films, successive deposition can be carried out following horizontal transferring and drying the film. It is recommended to obtain each layer from different spots on the trough surface [11]. Then, the completed samples need to be annealed at 120 °C, for at least 1 h. Further details of the deposition method and conditions are summarized in [23,57,58,59].

Compared with PVDF, P(VDF-TrFE) can be directly processed into ferroelectric phase after annealing at an appropriate temperature, which makes investigations of ferroelectric neat PVDF films rare. However, due to the inherent low Curie temperature and thermal stability, large-scale integration of P(VDF-TrFE) is hindered. Thermal treatment causes not only instability of the remanent polarization but also crystal growth and an increase in surface roughness. These factors are sure to diminish the robustness of ferroelectric microelectronic devices, such as unacceptable leakage currents [51]. For ultra-thin P(VDF-TrFE) LB film, similar phenomena forming nanomesas or nanoislands were also reported [60,61,62,63,64]. However, ultra-thin PVDF films can still remain continuous after the annealing process, and the corresponding prosperities have been verified by scanning probe microscopy (SPM), where the smooth morphology and writeable ferroelectric domains of LB PVDF films were examined [5].

## 3. Characterization

### 3.1. Fourier Transform Infrared (FTIR)

**Figure 5:**
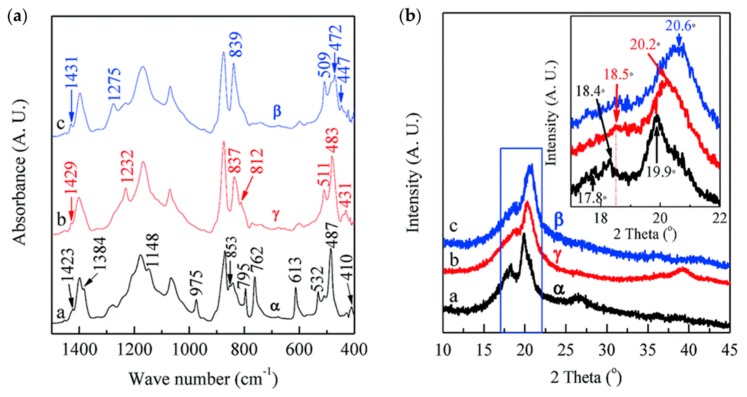
(**a**) FTIR absorbance spectra; (**b**) XRD diffractograms of PVDF membranes. Reproduced from [65], with permission from Royal Society of Chemistry, 2017.

FTIR spectroscopy of PVDF yields valuable information about its structure, allowing both qualitative identification and quantitative analysis of different crystal phases, which have been investigated thoroughly and extensively [18]. Although the assignment of some absorption bands remains disputable [65], there can be exclusive peaks for different polymorphs, mainly α, β, and γ phases. Figure 5a shows the general FTIR spectra of a PVDF membrane.

The α phase of PVDF can be easily obtained by recrystallizing PVDF from melt at any temperature; therefore, the exclusive absorption bands of this phase can be easily detected [18]. The characteristic spectrum of α phase is at around 410, 489, 532, 614, 766, 795, 854, 975, 1149, 1209, 1383, and 1423 cm^–1^ with small wavenumber shifts (typically within 2 cm^–1^), possibly deriving from experimental uncertainty [65,66,67,68,69]. The parallel version of α phase—δ phase—prepared by spin-coating was recently measured, and it showed a similar FTIR spectrum to α phase with the same absorption band locations, but different amplitudes [51].

Because of the similar chain conformation of β and γ phases, some measurement results of the respective absorption bands in the literature are contradictory. For instance, the bands at 510 and 512 cm^–1^ belong to β and γ phase, respectively [69]. Owing to the close location, it is often not easy to distinguish between the two bands. For the 840 cm^–1^ absorption band, it has been accepted that the band is common to both β and γ phases, but it is a strong band only for the β phase, whereas it appears as a shoulder of the 833 cm^–1^ band for the γ phase [70,71]. The absorption bands at 881, 1071, 1176, 1401, and 1431 cm^–1^ are also disputed [65]. Deconvolution is necessary in order to analyze the aforementioned overlapping bands. 

However, there are still some obviously exclusive bands for β phase at around 445, 473, and 1279 cm^–1^, while the 431, 482, 776, 812, and 1233 cm^–1^ bands correspond exclusively to the γ phase. Based on several unique bands of FTIR results, some methods for quantitatively analyzing the electroactive phases in samples have been developed. The method developed in [67] is widely used to calculate the percentage of β phase in films containing mainly α and β phase. Assuming that *A_α_* and *A_β_* are the respective absorbance at 766 and 840 cm^–1^, the following equations are available:

(1) $$A_{\alpha }=\log\frac{I_{\alpha }^{0}}{I_{\alpha }}=K_{\alpha }CX_{\alpha }t,$$

(2) $$A_{\beta }=\log\frac{I_{\beta }^{0}}{I_{\beta }}=K_{\beta }CX_{\beta }t,$$

(3) $$F\left(\beta \right)=\frac{X_{\beta }}{X_{\alpha }+X_{\beta }}=\frac{A_{\beta }}{(K_{\beta }/K_{\alpha })A_{\alpha }+A_{\beta }},$$

where *t* represents the sample thickness; and *C* represents the average total monomer concentration, which is a constant for a specific sample and can be determined by X-ray diffraction (XRD) patterns. The *α* and *β* subscripts refer to the two crystalline phases, *I^0^* and *I,* are the incident and transmitted intensity radiations, respectively, *K* is the absorption coefficient at the respective wavenumber, and *X* is the degree of crystallinity of each phase. The values of *K*_α_ and *K*_β_ were determined as 6.1 × 10^4^ and 7.7 × 10^4^ cm^2^/mol, respectively [67]. In some papers, the constant *C* is often multiplied by *K* to generate a new variable for the absorption coefficient with the unit of μm^–1^.

For films containing α, β, and γ phases, quantitatively analyzing the electroactive phases is more sophisticated and can be achieved by a similar approach. The details are described in [72].

In films containing mainly α and γ phases with little β phase, a similar equation is available:

(4) $$F\left(\gamma \right)=\frac{X_{\gamma }}{X_{\alpha }+X_{\gamma }}=\frac{A_{\gamma }}{(K_{\gamma }/K_{\alpha })A_{\alpha }+A_{\gamma }},$$

where *A_α_* and *A_γ_* represent the absorbance at 766 and 833 cm^–1^; and *K_α_* and *K_γ_* are the absorption coefficients at the respective wave numbers. The value of *K_α_* is 0.365 μm^–1^, while *K_γ_* is 0.150 μm^-1^ [67].

In addition to the FTIR spectra obtained by experimental measurements, the DFT calculations and the MDS were also utilized to analyze the vibrational properties of PVDF and P(VDF-TrFE) [14,15,17,73,74,75,76]. Wang et al. make detailed assignment for the vibrational spectra of α and β PVDF derived from DFT. The spectra were divided into five bands, corresponding to C–H stretching modes (Ι), C–H rocking modes (ΙΙ), C–H twisting and wagging modes (ΙΙΙ), –CH_2_ and –CF_2_ rocking and skeletal bending (ΙѴ and Ѵ), respectively [14]. Except for excellent agreement with the experimental results, the calculations indicate that peaks at 1347, 930, 463, and 449 cm^–1^ are potential references for further identification of the β phase of PVDF. Furthermore, Correia et al. compared the infrared peaks of defective and defect-free PVDF molecular chains of α and β conformation, and found that the presence of monomer inverted defects not only shifts, splits, and disappears existing peaks in the defect-free chains, but also gives rise to new peaks [17,75]. The conclusion indicated that the splitting of infrared peaks observed experimentally could be related to the presence defects in polymer chains rather than the presence of different phases. Li et al. calculated the vibrational modes of β chains of PVDF according to the first principles and deduced that only the vibrational modes between 1330 and 1020 cm^–1^ were responsible for the spontaneous polarization according to their results [74].

### 3.2. XRD

According to the aforementioned FTIR spectrum, it is easy to differentiate α phase from β and γ phases, but the β and γ phases can be easily confused. XRD results can work as a supplement for FTIR to distinguish β and γ phases [4]. Although studies have proposed and validated general procedures for phase identification based only on FTIR results [65], XRD is still worthy of mention here, as shown in Figure 5b.

Similar to FTIR, the XRD results have also been thoroughly studied. The XRD pattern is summarized below, with 2θ ranging from 10° to 45°.

The α phase presents stronger diffraction peaks at 2θ = 17.66°, 18.30°, 19.90°, and 26.56°, corresponding to (100), (020), (110), and (021) diffractions planes, respectively [69,77,78]. There are also four weak peaks at 33.2°, 35.9°, 38.8°, and 41.1° corresponding to (130), (200), (002), and (111) reflections of the monoclinic α phase, respectively [79,80,81].

Upon the successful preparation of δ-phase neat PVDF, identification of the diffraction peaks was carried out, and results proved that the samples showed the same diffraction peaks as α phase, but the amplitude differed [51].

For β phase, it has been reported that a well-defined strong peak at 2θ = 20.26° is related to the total diffraction at (110) and (200) planes [69,78], whereas some researchers argue that a strong diffraction peak at 20.6° and a weak peak at 36.3° represent β phase [78,80,81].

For γ phase, a superposition of peaks at 18.5° and 19.2° is associated with (020) and (002) planes. Some works have reported that a more intense peak at 2θ = 20.04° corresponds to the (110) crystalline plane, while the close peak at 20.3° is attributed to (110/101) planes. Besides, γ phase was found to have a weak peak at 26.8° attributed to the (022) plane and a peak at 39.0° for the (211) plane [18].

### 3.3. Ferroelectric Capacitor

A ferroelectric capacitor can be fabricated by sandwiching a ferroelectric thin film between two electrodes, which is a commonly used device for obtaining the physical characteristics of the corresponding ferroelectric materials. 

**Figure 6:**
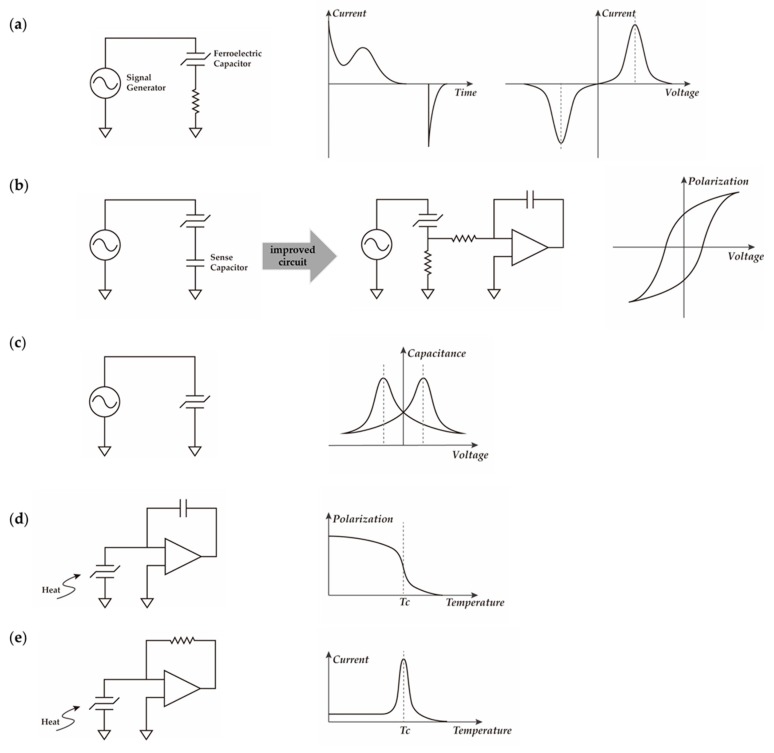
Circuit for characterizing ferroelectric thin films: (**a**) *I* vs. *t* and *I* vs. *V*; (**b**) *P* vs. *V*; (**c**) *C* vs. *V*; (**d**) *P* vs. *T*; (**e**) *I* vs. *T*.

Figure 6 shows each of the typical test circuits and corresponding results. Figure 6a illustrates the *I–t* curve at impulse voltage and the *I–V* response of ramp voltage. The switching and non-switching currents can be determined from the *I–t* curve, while the value of coercive voltage can be acquired from the *I–V* curve. The basic Sawyer–Tower circuit for hysteresis loop measurement is shown in Figure 6b, and a virtual ground is often added to avoid noise, as presented in the improved circuit. In Figure 6c, with a small signal superposed DC voltage bias, the circuit is used to measure the *C–V* curve. Temperature-related properties such as the Curie point can be determined by the circuits shown in Figure 6d,e [82].

There have been numerous papers about ferroelectric capacitors based on PVDF and its copolymers. Bune et al. observed a first-order ferroelectric phase transition with a transition temperature nearly equal to the bulk value in capacitors of ultra-thin P(VDF-TrFE) LB films. Moreover, they claimed that the thickness of these ultra-thin ferroelectric films reached the two-dimensional level [55]. Based on the experimental results of the corresponding capacitor, several groups have given different views on the switching dynamic of PVDF and P(VDF-TrFE) [83,84,85]. Polarization switching kinetics can also be studied by means of such ferroelectric capacitors [86,87,88,89,90,91,92].

In the published literatures, a number of different materials have been deposited as the electrodes, including aluminum, platinum, gold, silver, copper, nickel, tungsten, and indium tin oxide (ITO). It seems that the choice of electrodes can influence the result. After the phenomenon of intrinsic switching in ultra-thin P(VDF-TrFE) LB films reported by Ducharme et al. [84], some researchers ascribe it to the electrode interfaces [83]. Among those metals, aluminum is popular for the advantages of low deposition temperature and low work function. However, an Al_2_O_3_ insulator layer may form between the ferroelectric film and the electrodes, affecting the result [93].

While testing ferroelectric capacitors, size effects have an important influence on the properties of ferroelectric films, such as coercive field and remanent polarization. The details of polarization switching properties degradation caused by shrunken thickness and lateral film size are presented elsewhere [1,82].

### 3.4. Scanning Probe Microscope (SPM)

SPM is a general term for several probe-related microscopes, such as atomic force microscope (AFM), electrostatic force microscope (EFM), and Kelvin probe force microscope (KFM) [94]. These technologies have been used to study ferroelectric materials [95].

The piezoresponse force microscope (PFM) is a voltage-modulated version of AFM, and after Güthner et al. used it to research the local poling of ferroelectric P(VDF-TrFE) film in 1992 [96], it took several years for PFM to become a mainstream analytical tool of ferroelectrics. PFM is able to image and manipulate ferroelectric domains nondestructively, and the physical properties (e.g., domain wall dynamics, bias of nucleation, piezoelectric coefficients, and coercive voltage) can be measured directly [97]. There have been many books and papers describing the technical details of PFM comprehensively [94,95,98,99,100,101], and it is worth noting that the researchers need to understand the principle of PFM in order to avoid misinterpreting the data and drawing incorrect conclusions [97].

Aside from ferroelectricity, there can be several other factors causing ferroelectric-like PFM response. Thus, it is essential to verify the existence of ferroelectricity by detecting domains in diverse polarization directions and switching the domain states hysteretically under applied electric fields [94,97].

## 4. Physical Theory

In order to commercialize a material successfully, a predictive and robust understanding of the materials and related underlying mechanisms is necessary. For PVDF and its copolymers, the crystal structures and phase transitions have been studied in depth since the 1970s, and several significant books have reviewed this topic [11,13]. Further research on the polarization switching dynamics has also been reported. Due to the great potential of ferroelectric PVDF and its copolymers, it is important to keep the polarization switching dynamics in mind, which will guide how to intentionally design with these materials and how fast the devices can work [90].

**Figure 7:**
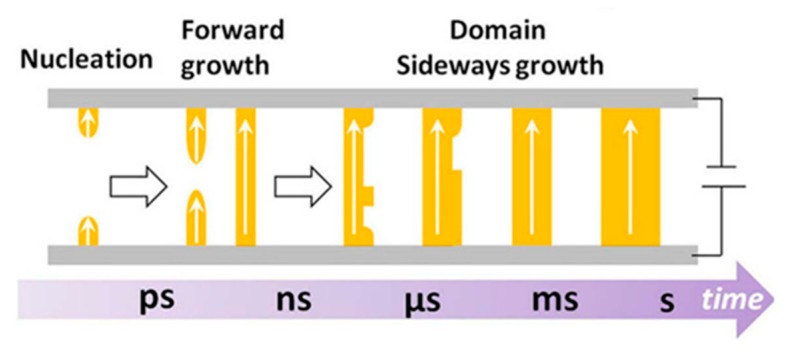
General process of polarization switching. Reproduced from [90], with permission from Springer Nature, 2014.

In the study of ferroelectric barium titanate (BaTiO_3_), Merz [102,103], Little [104], Miller [105,106], and Fatuzzo [107] established the universal mechanism of polarization switching, which is generally expressed in three steps: nucleation, forward domain growth, and succedent sideways growth. Firstly, an electric field with opposite polarity to ferroelectric polarization stimulates the small nuclei of the domain to form at the grain boundaries or interfaces. Then, each nucleus grows forward until it reaches the opposite side and becomes a domain. Finally, domains grow sideways and start to unite. This continuous process does not stop until the polarization is completely reversed in the whole sample, as shown in Figure 7 [93].

To date, there have been several detailed models proposed to quantitatively explain the mechanism of domain-derived polarization switching dynamics. Originally, in the Kolmogorov–Avrami–Ishibashi (KAI) model, the homogeneous formation of numerous nucleations and subsequent forward and sideways domain growth was assumed [89,90,108,109]. Next, Tagantsev et al. put forward the nucleation-limited switching (NLS) model as an alternative model for the polarization switching process [86], in which the region-by-region nucleation and switching take much longer than the domain wall motion. While studying ultra-thin PVDF and P(VDF-TrFE) films, a domain-free intrinsic mechanism that did not include the ordinary nucleation and domain growth processes was developed [84,110,111]. Here, the three previously mentioned theoretical models of ferroelectric switching are summarized.

### 4.1. KAI Model

The KAI model assumes homogeneous nucleation and unlimited domain growth in an infinite media, so it imprecisely matches the polarization switching in ferroelectrics with limited size and polycrystalline films/bulks. However, it is practicable for uniformly polarized single crystals and epitaxial films [89,112,113]. The model states that the reversed polarization *P*(*t*) and the switching current density *I*(*t*) can be denoted as follows:

(5) $$P\left(t\right)=2P_{s}\left\{1-\exp\left[-{\left(\frac{t}{t_{0}}\right)}^{n}\right]\right\},$$

(6) $$I\left(t\right)=2P_{s}\frac{n}{t_{0}}{\left(\frac{t}{t_{0}}\right)}^{n-1}\exp\left[-{\left(\frac{t}{t_{0}}\right)}^{n}\right],$$

where *P*_s_ stands for spontaneous polarization, *t*_0_ is the characteristic switching time, and *n* is a parameter valued between 1 and 3, describing both the rate of nucleation and the dimension of subsequent growth. There is no physical meaning for *n* less than 1. 

To date, the KAI model has been reported to perfectly explain the polarization switching in many inorganic ceramics [89]. Hu et al. recently declared that their data suggest that the polarization reversal in P(VDF-TrFE) films could also be well modeled by the classic KAI mechanism, similar to ceramic counterparts with a fast-switching process [90].

### 4.2. NLS Model

Due to the abovementioned limitation of the KAI model, the NLS model was subsequently constructed as an alternative scenario in [86]. This model assumes that the film consists of many regions that nucleate and switch independently, and it well suited for explaining the switching processes that need much more time in some ferroelectric thin films, where the time of nucleation is much longer than that of domain wall motion [86,114,115]. In consideration of the ensemble of the elementary regions, the expression of time-dependent polarization in the KAI model is upgraded with a distribution function of the switching times, written as follows [86]:

(7) $$P\left(t\right)=2P_{s}\int_{-\infty }^{\infty }\left\{1-\exp\left[-{\left(\frac{t}{t_{0}}\right)}^{n}\right]\right\}F\left(\log t_{0}\right)d\left(\log t_{0}\right),$$

where $F\left(\log t_{0}\right)$ is the distribution function of $\log t_{0}$. It should meet the normalizing condition:

(8) $$\int_{-\infty }^{\infty }F\left(\log t_{0}\right)d\left(\log t_{0}\right)=1.$$

The value of *n* is assumed to be 2 for thin-film ferroelectrics and 3 for bulk ferroelectrics. Jo et al. and Zhukov et al. respectively suggested that the Lorentzian distribution and Gaussian distribution of $\log t_{0}$ could be applicable for the random electric field distribution [87,91]. Mao et al. took advantage of the NLS model to fit the independent region-by-region polarization reversal in a polycrystalline P(VDF-TrFE) (100 nm) thin film, and they found that the model described the polarization reversal behavior in the time domain well [88].

### 4.3. Intrinsic Switching

**Figure 8:**
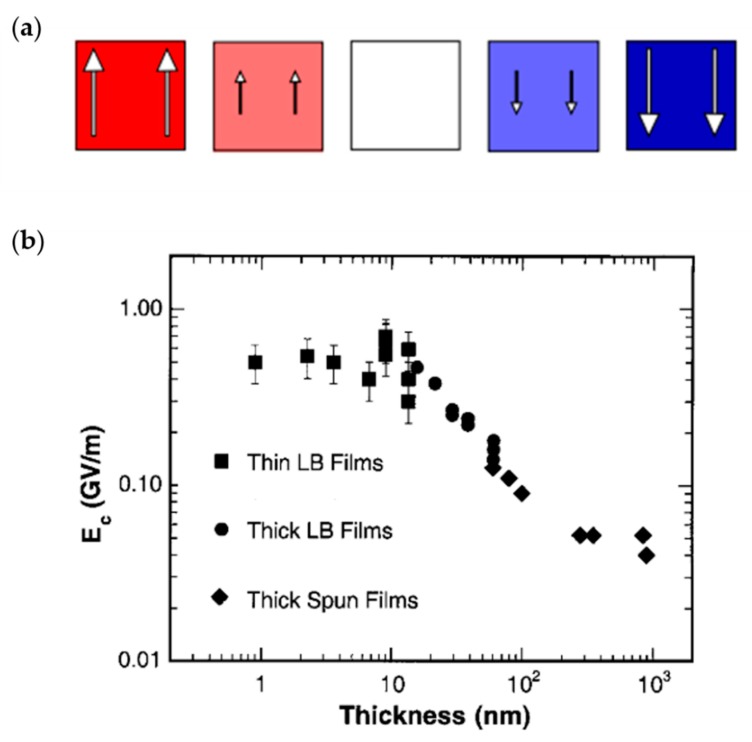
(**a**) Polarization switches uniformly without domain formation. (**b**) The thickness-dependent coercive field of P(VDF-TrFE). Reproduced from [84,118], with permission from American Physical Society, 2010 and 2000, respectively.

The studies of ultra-thin PVDF and P(VDF-TrFE) films prepared by LB method suggest another switching dynamics that differs from nucleation and domain wall motion [84,116]. Both the KAI model and the NLS model assume a sufficient volume for nucleation and domain growth. Therefore, they are not adequate to explain the switching dynamics in ultra-thin films [117]. Thus, based on the Landau–Ginzburg–Devonshire (LGD) theory, a polarization reversal phenomenon named intrinsic (homogenous) switching was put forward and is illustrated in Figure 8a.

According to the LGD theory, there is an intrinsic coercive field *E*_c_ of an ultra-thin ferroelectric film, which means that upon applying the electric field *E* < *E*_c_, no polarization switching should occur, only *E* > *E*_c_ can activate the switching process because the highly correlated dipoles in the crystal tend to switch coherently or not at all [84,85,117,119,120].

In the absence of nucleation, the expected value of *E*_c_ is enormous, on the order 100 MV/m in most ferroelectrics [85]. Ducharme et al. observed the existence of intrinsic switching in P(VDF-TrFE) LB films for the first time. They suggested that the coercive field of P(VDF-TrFE) LB films increases with decreasing thickness at first and then saturates for samples with thickness below a critical value (15 nm), at a magnitude of about 500 MV/m [84], as shown in Figure 8b. Note that, when the intrinsic switching dominated, the coercive voltage was not more than 5 V because of the extreme small thickness of films. Recently, Paramonova et al. simulated the intrinsic polarization switching process of PVDF and P(VDF-TrFE) by using the MDS method, and they demonstrated a coercive field lying within 500–2500 MV/m and a critical thickness of 3–6 nm [111].

Staring from the Landau–Khalatnikov equation, the phenomenon of intrinsic switching is explained as follows:

(9) $$\xi \frac{dP}{dt}=-\frac{\partial G}{\partial P},$$

where ξ and *P* are the polarization damping coefficient and the spontaneous polarization, respectively. The Gibbs free energy *G* is assumed to be a function of the spontaneous polarization *P*, external electric field *E*, and temperature *T*, on the basis of the LGD theory. 

**Figure 9:**
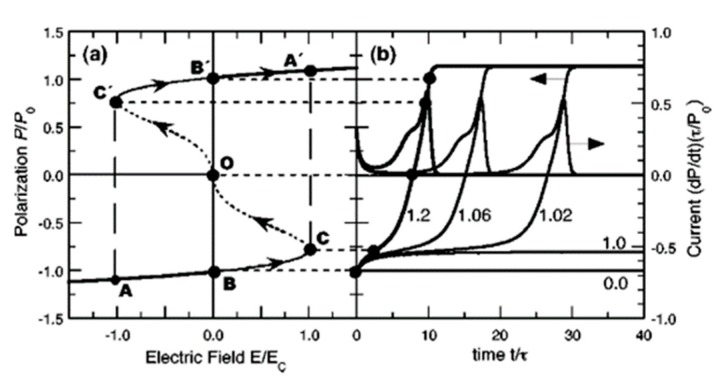
(**a**) Normalized *P*(*E*) hysteresis loop derived from Equation (9), with stable, metastable, and unstable states denoted in heavy, thin, and dotted lines, respectively. (**b**) Evolution of the normalized polarization over time and current during switching. Reproduced from [119], with permission from American Physical Society, 2003.

As shown in Figure 9a, a normalized steady-state *P*(*E*) hysteresis loop is obtained by minimizing the Gibbs free energy. With the heavy curves (AB and A’B’) denoting stable states, thin curves (BC and B’C’) denoting metastable states, and the dotted curve (COC’) denoting an unstable state, applying an eternal electric field $E<E_{c}$, the polarization will not cross the unstable state to another stable state, and it returns to its initial stable state from the metastable state [110,119].

By solving Equation (9), the switching time *τ*, defined as the time needed by the polarization to change from a stable state to zero, is depicted in Figure 9b. It is obvious that if *E* is not more than *E_c_*, the switching time *τ* tends to infinity, which mean that the polarization could not be reversed [110].

After observing the intrinsic switching in ultra-thin P(VDF-TrFE) LB films, and in order to avoid the influence of electrode, Gaynutdinov et al. gave new experimental results, with the help of PFM, to support that the intrinsic switching mechanism does exist at the nanoscale [121,122]. In addition, Tian et al. observed intrinsic switching in pure PVDF ultra-thin films [116]. Later, intrinsic switching was also found in ultra-thin epitaxial PbTiO_3_ films and ultra-thin ferroelectric BaTiO_3_ films [118,123].

## 5. Application

### 5.1. Nonvolatile Memory

**Figure 10:**
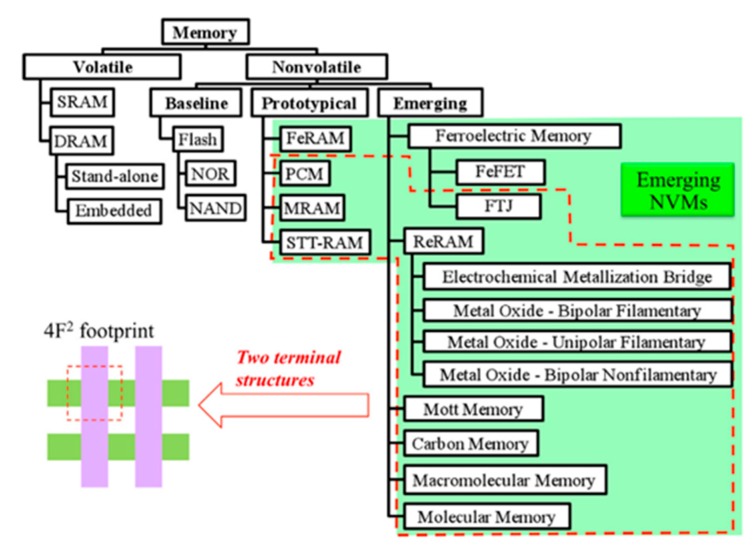
Memory taxonomy. Many emerging nonvolatile memories (NVMs) are in simple two-terminal devices and suitable for high-density crossbar arrays. Reproduced from [128], with permission from Elsevier, 2016. SRAM: static random access memory; DRAM: dynamic random access memory; PCM: phase change memory; MRAM: magnetic random access memory; STT-RAM: spin torque transfer random access memory; FeFET: ferroelectric field effect transistor; ReRAM: resistive random access memory; FTJ: ferroelectric tunneling junction.

One of the most important applications of PVDF and its copolymers is nonvolatile memory [124]. Memory is expected to possess high speed, energy efficiency, reliability, integration capabilities, and low cost. Over the past few decades, flash memories, dynamic random access memories (DRAMs), and hard-disk drives (HDDs) have occupied a large part of the memory market with all-around performance [125]. However, each of these three mainstream memories has shortcomings: flash memories suffer from low write and read speeds and a limited number of rewrite cycles; DRAMs are volatile and require frequent refreshes; and HDDs respond slowly to magnetic fields [126]. In order to explore potential memory devices, various mechanisms (e.g., magnetic effects, electrostatic effects, and atomic-configuration-based effects) have been extensively studied, and a large number of corresponding devices have been proposed [127,128]. Figure 10 shows the exploration of new a device for a next-generation memory cell.

**Figure 11:**
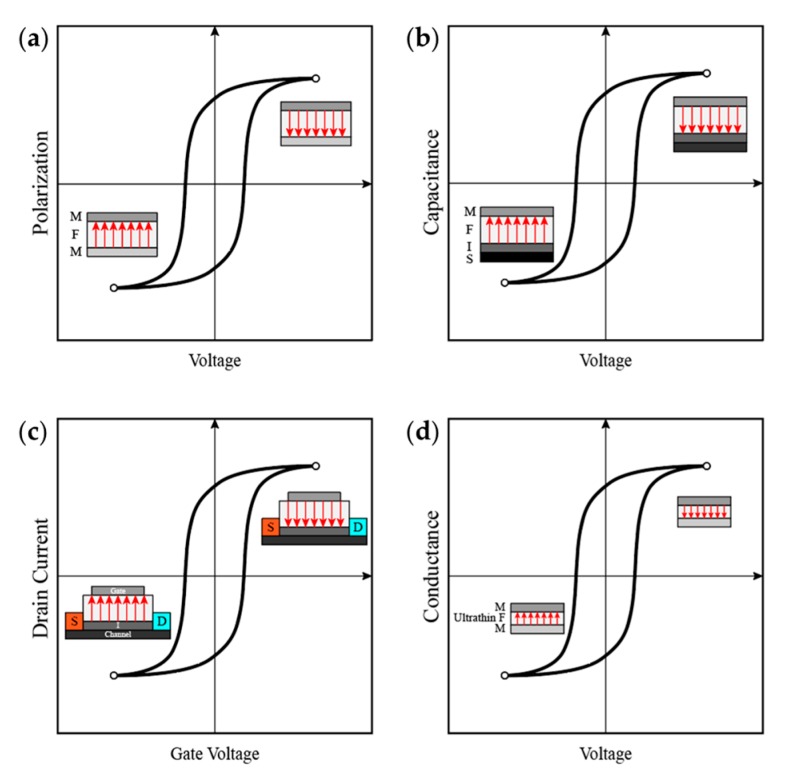
Schematic of different ferroelectric memories: (**a**) polarization–voltage hysteresis of a metal–ferroelectric–metal (MFM) capacitor; (**b**) capacitance–voltage hysteresis of a metal–ferroelectric–insulator–semiconductor (MFIS) diode; (**c**) drain current–gate voltage hysteresis of FeFET; (**d**) conductance–voltage hysteresis of FTJ.

Ferroelectric memories store the information by ferroelectric polarization, which can be reversed by an external electric field that exceeds the coercive field. Even if the electric field is removed, the remanent polarization can still maintain state and retain information [129]. For convenience, they are broadly classified into four categories: metal–ferroelectric–metal (MFM) capacitors, metal–ferroelectric–insulator–semiconductor (MFIS) diodes, ferroelectric field effect transistor (FeFET), and the emerging ferroelectric tunneling junction (FTJ). Figure 11 briefly shows their working principles.

**Figure 12:**
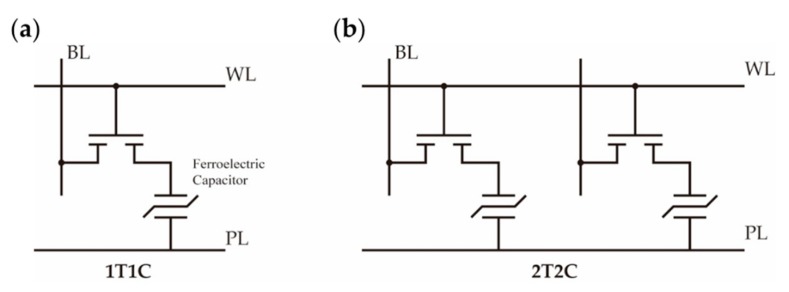
The ferroelectric memory cell of an MFM capacitor and transistor: (**a**) 1T1C (one-transistor–one-capacitor); (**b**) 2T2C (two-transistor–two-capacitor). BL (bit line), WL (word line), and PL (plate line).

For the first category, the memory cell can be formed by connecting an MFM capacitor and a transistor in a 2T2C (two-transistor–two-capacitor) or 1T1C (one-transistor–one-capacitor) structure [130], as shown in Figure 12. In the early stages of development, a self-referencing 2T2C cell was adopted to ensured stable access operation; later, a 1T1C cell was introduced and shrank the required area by nearly half [1]. However, 1T1C cells operate in a destructive read-out manner, and thus the data have to be restored by a rewriting cycle after readout.

The MFIS diode is another type of memory whose capacitance is controlled by the voltage on the metal electrode. This type of memory shows a capacitance hysteresis between read–write voltage margins. Among PVDF and its copolymers, P(VDF-TrFE) made with the aforementioned spin-coating or LB deposition methods is the most reported material for fabricating MFIS diodes [131,132,133,134,135,136,137,138,139,140]. Reece et al. prepared a device consisting of a top metal electrode, an LB film of P(VDF-TrFE) (170 nm), and a SiO_2_ (100 nm) insulating layer on an n-type Si substrate. While the gate voltage was swept between −25 and +25 V, a clear *C–V* hysteresis was measured [131]. The relatively thick ferroelectric film and insulating layer led to high working voltage, so the thickness was reduced in the following work. Gerber et al. deposited a P(VDF-TrFE) film (36 nm) on thermally oxidized p-type Si (10 nm SiO_2_), in which *C–V* hysteresis could be obtained at the sweeping voltage of ±3 V [133]. Fujisaki et al. used Ta_2_O_5_ instead of SiO_2_ as an insulating layer and deposited P(VDF-TrFE) (100 nm) by spin-coating. The voltage sweep range could be decreased to no more than ±5 V to obtain a rectangular *C–V* hysteresis [135].

**Figure 13:**
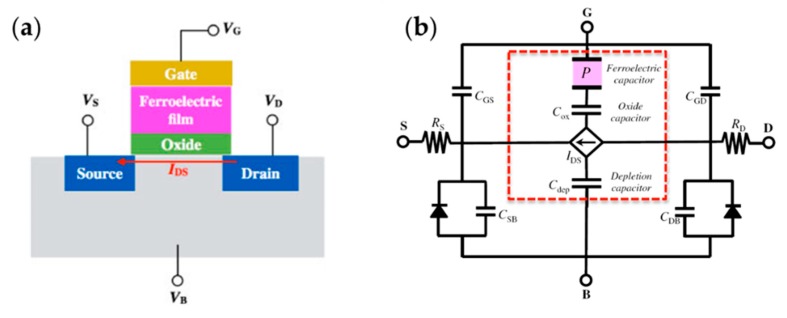
FeFET with metal–ferroelectric–insulator–semiconductor (MFIS) structure: (**a**) schematic of the FeFET; (**b**) equivalent circuit model of the FeFET. The dotted square indicates the gate-to-channel region of the FeFET. Reproduced from [148], with permission from IOP publishing, 2017.

FeFETs have a fundamental structure of metal–ferroelectric–semiconductor (MFS), and an insulator is often sandwiched between the semiconductor and the ferroelectric, forming an MFIS structure to improve the tolerances to severe conditions during the device preparation and to avoid charge injection into inorganic ferroelectrics [2], as shown in Figure 13. Although they have a similar structure to MFIS diodes, FeFETs work by utilizing ferroelectric polarization to modulate the channel conductance and differentiate the two logic states in a nondestructive manner [141,142]. In the past reports, there have been attempts to fabricate FeFETs with ferroelectric P(VDF-TrFE), PVDF, and P(VDF-TeFE) [2,143,144,145,146,147]. Naber et al. reported high-performance solution-processed FeFETs consisting of a P(VDF-TrFE) gate dielectrics and MEH-PPV (poly[2-methoxy,5-(2΄-ethyl-hexyloxy)-p-phenylene-vinylene]) as the semiconductor. The device showed short programming time, long data retention, high programming cycle endurance, and high operation voltage [144]. Park et al. reported FeFETs with MFS structure fabricated by depositing films of PVDF or its copolymers on a silicon substrate. Without the insulating buffer layer, the depolarization field was eliminated, the structure was simplified, and, moreover, the proposed device could work under even lower operation voltage (i.e., less than 1 V) [2].

**Figure 14:**
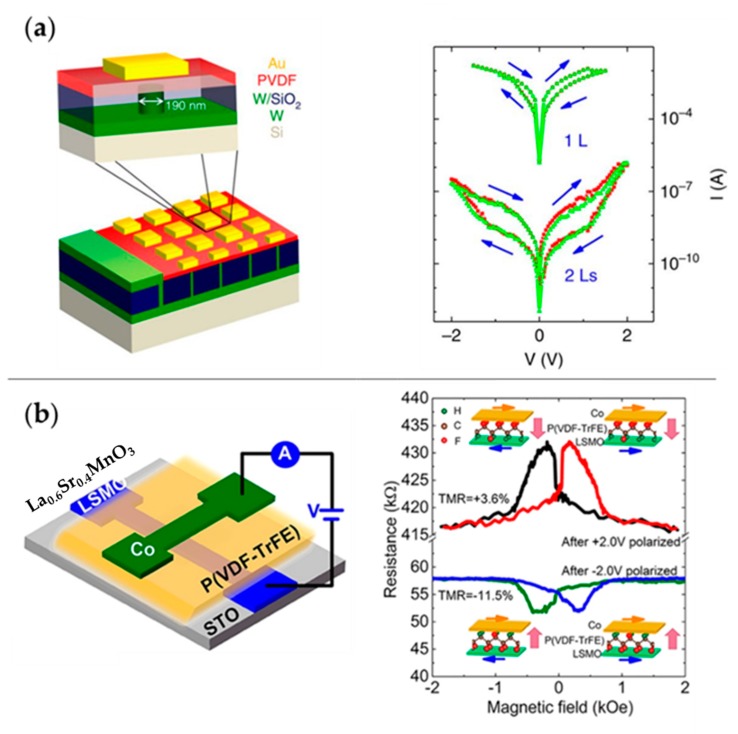
FTJ and multiferroic tunnel junction (MFTJ) based on PVDF or P(VDF-TrFE): (**a**) schematic of PVDF FTJs and the *I–V* curves in the 1- and 2-layer devices; (**b**) schematic of the MFTJ of LSMO/P(VDF-TrFE)/Co on SrTiO_3_ (STO) substrate and measured magneto-response curves. Reproduced from [5,157], with permission from Springer Nature, 2016 and American Chemical Society, 2018, respectively. TMR: tunnel magnetoresistance.

In addition to the aforementioned devices, the FTJ structure that was theoretically proposed by Esaki in 1971 [149] is another emerging promising nonvolatile memory. Figure 14a shows an FTJ based on PVDF LB films. The concept was experimentally demonstrated to be available in the 2000s, with the improvement of high-quality thin-film preparation and characterization techniques [150,151]. Several years later, the phenomenon of resistive switching was unambiguously correlated to the ferroelectricity of the material used, and the relevant theory was developed [152]. The electronic transport interacts with ferroelectricity in a rather complex way, but the main mechanism producing giant tunnel electroresistance (TER) in FTJs is that the asymmetric electronic potential profile induced by polarization charge effects has an average barrier height that changes with the configuration of ferroelectric polarization [153,154,155]. Because the tunnel transmission depends exponentially on the square root of the barrier height, the junction resistance depends on the direction of polarization. According to this classic electrostatic model, the tunneling current strongly depends on the electronic properties of the electrode/ferroelectric interfaces in FTJs; as a result, a higher OFF/ON resistance ratio can be achieved by choosing the electrode materials carefully [156].

Most proposed FTJs are based on inorganic materials [151,158,159,160,161]. Qu et al. studied 2-monolayer P(VDF-TrFE) LB films and gave evidence of conductance switching at the nanoscale with local polarization reversal [162]. Tian et al. demonstrated solid-state nanodevices based on a ferroelectric LB PVDF ultra-thin film, and a resulting TER of >1000% at room temperature was measured [5]. Kusuma et al. successfully fabricated and characterized organic FTJ devices, in which vinylidene fluoride (VDF) oligomer (a short-chain version of PVDF) served as the intermediate ferroelectric layer. Despite the modest ON/OFF current ratio, the device exhibited low switching voltages (±1.0 V), excellent cycling stability (10^4^ cycles), and excellent data retention (10^3^ s) [163].

A great deal of development is still required before PVDF and its copolymers can be applied in practical FTJ memories. Performance metrics such as energy efficiency, OFF/ON ratios, endurance, data retention, and scalability urgently need to be optimized.

Ferroelectricity and ferromagnetism show many similar features. Analogous to the TER effect in FTJs, the tunnel magnetoresistance (TMR) effect exists in magnetic tunnel junctions (MTJs), in which an ultra-thin dielectric layer is sandwiched between two ferromagnetic electrodes [164,165]. Controlled by a magnetic field, the parallel and antiparallel configurations of the magnetic electrodes correspond to two distinct tunnel resistances of these devices [156]. Thus, the ferroelectricity and ferromagnetism can be coupled in a multiferroic tunnel junction (MFTJ) that sandwiches a ferroelectric tunnel barrier between two ferromagnetic electrodes. Figure 14b shows the P(VDF-TrFE)-based MFTJ. The MFTJs can generate four distinct resistance states owing to the coupling of inherent TER and TMR effects [155]. Since Gajek et al. first demonstrated MFTJ to be a memory with four resistance states in 2007 [166], the study of MFTJ has been booming [167,168,169,170]. 

Lopez-Encarnacion et al. reported a simulation of employing PVDF as barriers in MFTJs and showed that the combination of the ferroelectric polarization orientations of the barrier and the configurations of electrodes produced multiple conductance states in the devices [171]. Recently, there have been solid-state MFTJs prepared based on PVDF or its copolymers [157,172].

### 5.2. Memristive Devices and Artificial Neutral Synapse

A memristor (a contraction of “memory-resistor”) is a two-terminal device whose conductance can be tuned by the history of applied voltage and current [173]. It was theoretically conceived and mathematically formulated in the 1970s [174,175], and it was subsequently linked to resistive switching devices in 2008 [176]. In the past two decades, memristor technology has attracted considerable interest, and various memristive devices have been reported to be competitive candidates for neuromorphic systems [177,178,179,180,181,182,183], in-memory logic [184,185,186], and analogue computing [187].

In a neuromorphic systems, synapses are realized by memristive devices, whose tunable conductance fits well with the synaptic weight in biologically plausible learning rules such as spike-timing-dependent plasticity (STDP) [188]. The desirable performance metrics for organic memristive devices are summarized in Table 2 [189]. In general, the more multilevel states a single device reflects, the better the learning capability that improves the robustness of a network after training. Grouping multiple devices with fewer tunable levels can also achieve higher accuracy, but at the expense of energy and area [190,191].

FTJs have been demonstrated to be members of memristors [192,193]. In the past few years, FTJs with various ferroelectric materials (e.g., PVDF [5], BTO [160], PZT [159], and BFO [194]) were reported, and the physical mechanisms inducing TER also gained further understanding [195]. It is theoretically possible to exploit nonuniform ferroelectric domains, to build a multistate between the highest and lowest resistance states corresponding to the opposite directions of the ferroelectric polarization [193]. Recently, there have also been attempts to apply FTJ synapses in neural networks [196,197]. However, an FTJ based on a PVDF LB film was demonstrated to have an OFF/ON ratio of >1000% at room temperature [5], which is still not sufficient. Recently, Majumdar et al. reported organic FTJ memristors for neuromorphic computing. With solution-processable P(VDF-TrFE) copolymer as a barrier, the FTJ behaved as an energy-efficient analog memristor with a broad range of accessible conductance states [198].

A FeFET is also useful as artificial neural synapse [1]. In this application, the amplitude or duration of an input pulse signal can be adjusted to manage the polarization of the ferroelectric film; thus, the channel conductance can be tunable. Recently, Tian et al. reported a robust FeFET-type artificial neural synapse based on a spin-coated P(VDF-TrFE) film. Due to the interplay of ferroelectric domains and MoS_2_ channel, the conductance of this device can be precisely manipulated at the highest OFF/ON ratio of ≈10^4^, with more than 1000 intermediate states [9].

### 5.3. Flexible Electronic Devices

Compared with rigid and fragile inorganic materials, PVDF and its copolymers are wildly exploited in flexible electronic devices, mainly energy harvesters, self-powered electronics, and piezoelectric/pyroelectric sensors. Thin films required in these applications were usually fabricated by existing processes, such as spin-coating [199,200,201,202], solution casting, and electrospinning [203]. Recently, new technologies, such as thermal imprinting [204], nanoimprinting lithography, and photolithography [205,206], were also developed for patterning the prepared films. However, as the most convenient method, the spin-coating method is worth studying, and related remarkable works of applying spin-coated films in the abovementioned categories of the flexible electronic devices are worth reviewing.

**Figure 15:**
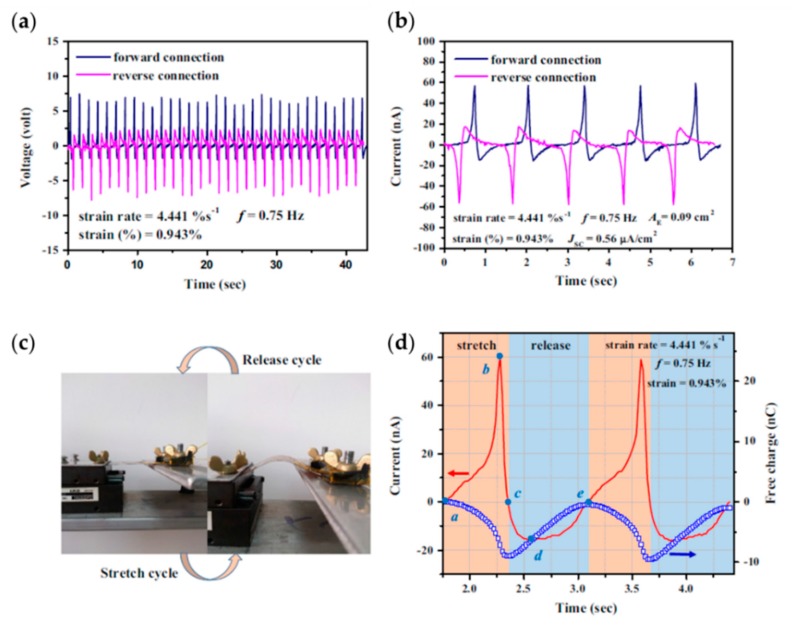
Characterizing the P(VDF-TrFE)-based nanogenerator: (**a**) the open-circuit voltage; (**b**) the short-circuit current; (**c**) equipment and connection for generating mechanical load to the nanogenerator.; and (**d**) analysis of stretching–releasing cycle. Reproduced from [201], with permission from Elsevier, 2014.

Energy harvesters provide a solution to the urgent demands of portable and renewable energy sources by the rapidly evolving technologies, such as mobile communications and personal care. Since the report of piezoelectric zinc oxide nanowire arrays serving as energy harvester [207], many structures of PVDF-based energy harvester have been explored to improve the energy conversion efficiency. Chen et al. introduced a novel energy-harvester design comprising gold nanoparticles modified electrode and spin-coated P(VDF-TrFE) film, and they demonstrated an enhanced power density of 1.8 mW/cm^3^, due to the increased effective area for charge collection [199]. Toprak et al. put forward a potentially complementary metal–oxide–semiconductor (CMOS) compatible fabrication by using spin-coated P(VDF-TrFE) film in microelectromechanical system (MEMS) scale cantilever-type piezoelectric energy harvesters. After applying a sinusoidal excitation causing 500 μm peak tip displacement, they measured an output power density of 97.5 pW/mm^2^ in the device [200]. Pi et al. measured an open-circuit voltage of 7 V and a short-circuit current of 58 nA, with current density of 0.56 μA/cm^2^ in spin-coated P(VDF-TrFE) film, as shown in the Figure 15. Further, an analytical model was developed for an as-prepared energy harvester [201]. Hu et al. demonstrated good stability in a double-layered structure of BT(nanocomposite film of BaTiO_3_ and PVDF)/PVDF(film of neat PVDF), and the output voltage and current of the device were 6.7 V and 2.4 μA, respectively [202].

Taking advantage of the piezoelectricity and pyroelectricity of PVDF-based polymers, pressure and temperature sensors were developed. It is inspiring that the spin-coating method can be integrated with MEMS processes, which facilitate the design of innovative devices [208,209,210,211,212]. Toprak et al. made a comprehensive characterization of the P(VDF-TrFE) thin films in MEMS applications, including the dielectric constant, dielectric loss factor, and the dependence of the ferroelectric responses on frequency and temperature [208]. Sharma et al. used a standard lithography process to pattern the spin-coated P(VDF-TrFE) film (1 μm), which was sandwiched between Al and Cr/Au electrodes. They declared that the device was suitable for measuring the real-time pressure in catheter applications, for inherent fast recovery time (0.17 s) and broad working range (0 to 300 mmHg) [209]. Dahiya et al. developed arrays of microelectrode and piezoelectric oxide semiconductor field effect transistor (POSFET) to mimic the sense of touch in humans [210,211]. As for the pyroelectricity, Viola et al. fabricated a flexible device that couples a charge-modulated organic field-effect transistor (OCMFET) with a P(VDF-TrFE) film for temperature sensing (8 to 50 °C) [213].

### 5.4. Medical Applications

As a biocompatible material with excellent chemical stability, PVDF is useful in many medical applications that can take advantage of the pyroelectricity and piezoelectricity of PVDF and its copolymers, including human physiological signals detection [214,215,216,217], tissue engineering scaffolds [218,219,220,221,222,223], and antifouling ultrafiltration membranes [224,225,226,227,228].

The main components are the sensors and the actuators used for detecting human physiological signals, such as temperature and pulse waves. Tien et al. constructed a device array of pressure and temperature sensor pixels on top of a flexible platform, to mimic the functions of a human finger. Each pixel was a flexible FET with an organic semiconductor (pentacene) as the channel and a nanocomposite material (a mixture of BaTiO_3_ nanoparticles and P(VDF-TrFE)) as the gate dielectric. The device could respond to temperature and pressure in a distinguishable manner and kept high sensitivity, which gives it the potential application as electric skin [215]. Trung et al. fabricated flexible and transparent FETs, with a reduced graphene oxide (R-GO)/P(VDF-TrFE) nanocomposite channel as a sensing layer by spin-coating. The device was capable of detecting small temperature changes (0.1 °C) and was highly sensitive to human-body temperature [216]. Shin et al. employed a ZnO nanoneedle/PVDF hybrid film in a wearable and wireless pressure sensor that could monitor heart rate without distortion or time delay [214].

The piezoelectricity of PVDF and its copolymers has also drawn attention in tissue engineering applications [219,220,221,222,223]. In early studies, piezoelectricity was predicted to be a universal property of living tissue, and it may play an important part in certain physiological phenomena [229,230,231]. Thus, with the higher piezoelectric response among optional materials, PVDF and its copolymers have been fabricated into several typical morphologies, such as films, fibers, porous membranes, and 3D porous scaffolds for different applications in tissue engineering, mainly for bone, muscle, and nerve regeneration [222]. However, electrospinning is generally adopted in most reports about PVDF and its copolymers in tissue engineering. Ferreira et al. demonstrated that electroactive macroporous P(VDF-TrFE) films of different thickness could also be fabricated by spin-coating, and they showed the material’s potential for smart scaffolds in tissue engineering [218]. There are also works suggesting that cell–material interactions and thrombus formation can be solved by improving the hydrophobic surface of PVDF for better biocompatibility. Taking advantage of non-mammalian sources of collagen, Wang et al. produced bioactive PVDF with comparable cell–material interactions and hemocompatibility [223].

Finally, composites of PVDF with materials such as TiO_2_ and MXene to form antifouling ultrafiltration membranes have shown noteworthy performance. Teow et al. incorporated TiO_2_ nanoparticles into a spin-coated PVDF membrane via phase inversion and colloidal precipitation. The membrane showed high permeability, with superior retention properties to humic acid [224]. Rajavel et al. prepared Ti_3_C_2_Tx-coated PVDF membranes that could effectively inactivate *Escherichia coli*, thus preventing biofilm formation on the active membrane surfaces and showing high potential for antibiofouling [227].

## 6. Outlooks

Nowadays, the fields of nonvolatile memory, artificial neural networks, and flexible electronic devices are rapidly developing and are certainly the most influential research areas in the next few years. The unique ferroelectricity and flexibility of PVDF and its copolymers makes prototype devices based on them promising in commercial applications. Meanwhile, extensive research into the processes that can optimize the thin films is ongoing. The spin-coating method generally produces high-quality and thickness-controllable thin film through convenient and repeatable steps. Combining with lithography process, the applicable scope is further expanded. Besides, proper nanocomposite filters in the solution used for film preparation can enhance the film’s performance. Despite these advantages, the drawbacks of the spin-coating method are obvious: that post-treatments, such as annealing, stretching, and poling, are generally necessary to improve the ferroelectricity of prepared sample. As for the LB method, it is well-known for the ability of producing ultra-thin films of even several nanometers. PVDF-based films prepared in this method are occupied primarily in memories up to now. However, the film of several monolayers must be treated as a two-dimensional material, which could integrate and interact with other new functional materials. In addition to these two methods, electrospinning is popular and wildly used in preparing flexible energy harvester and sensors. Furthermore, patterning thin films into required forms is another tendency of processes based on thermal imprinting and nanoimprinting are developed.

With the help of relatively mature technologies for characterizing piezoelectric, ferroelectric, dielectric properties, and morphology of PVDF-based polymer films, underlying physics, such as the polarization switching mechanism, size effect, and domain wall, have been elaborated for designing new devices. Although PVDF and its copolymers are irreplaceable among organic ferroelectrics, their inorganic competitors, such as BaTiO_3_, LiNbO_3_, and Hf_0.5_Zr_0.5_O_2_, have higher spontaneous polarization. In particular, Hf_0.5_Zr_0.5_O_2_ is considered to be the most industry-relevant ferroelectric material that is lead-free and compatible with current semiconductor manufacturing techniques. Extensive research still needs to be performed, focusing on putting the PVDF-based materials into practical use.

## 7. Conclusions

A comprehensive introduction and analysis of PVDF and its copolymers prepared via spin-coating and LB methods are presented in this review. PVDF is a material that combines flexibility and electroactive responses, and significant achievements have been made toward understanding the underlying physical mechanisms and putting them to use; however, there are still details that need to be considered for some critical technologies. For the applications mentioned above (especially memories and artificial synapses), the spin-coating and LB methods are both suitable for mass production, due to the cheap raw materials and facile processes. For medical applications, the processes of PVDF and its copolymers are not limited to spin-coating and LB methods; by adopting proper approaches, one can take full advantage of the materials.

## Figures and Tables

**Table 1:** Comparison of the piezoelectric, the pyroelectric coefficients, and the spontaneous polarization different material systems.

Materials	|*p*_3_| 10^–6^ C/(m^2^K)	|*d*_33_| 10^–12^ C/N	*P*_s_ C/m^2^
PVDF	27.4 [20]	24–34 [21]	0.065 [22,23]
P(VDF-TrFE) bulk films	7.6–30.4 [24]	34 [25]	0.02–0.08 [24]
P(VDF-TrFE) LB films	16–24 [26]	18–22 ^1^ [26]	0.1 [27]
P(VDF-CTFE) ^2^	–	140 [28]	0.04 [29]
P(VDF-TeFE)	–	–	0.02 to 0.04 [2]
Nylon-11	–	–	0.05 [30]
BaTiO_3_	–	140 [31]	0.16–0.25 [32]
LiNbO_3_	–	6 [33]	0.46 [32]
Lantano-doped HfO_2_	–	–	0.45 [34]

^1^ The unit is 10^-12^ m/V. ^2^ Poly(vinylidene fluoride–chlorotrifluoroethylene).

**Table 2:** Desired and recommended metrics for organic neuromorphic devices. Reproduced from [189], with permission from Springer Nature, 2018.

Parameter	Value
Size for integration	<1 μm^2^ arrays for dense/compact
Number of states	~100 separable states, or ~6 bit
Conductance tuning	Linear and symmetric
Switching noise	<0.5% of weight range
Switching energy	<1 pJ per switching event
Write/read speed	<1 μs
State retention	10^3^–10^8^ s
Write endurance (cycles)	~10^9^ (online learning)
Temperature stability	Array operating temperature

## References

[1] Ishiwara H., Okuyama M., Arimoto Y. (2004). Ferroelectric Random Access Memories: Fundamentals and Applications.

[2] Park B.-E., Ishiwara H., Okuyama M., Sakai S., Yoon S.-M. (2016). Ferroelectric-Gate Field Effect Transistor Memories: Device Physics and Applications.

[3] Setter N., Damjanovic D., Eng L., Fox G., Gevorgian S., Hong S., Kingon A., Kohlstedt H., Park N.Y., Stephenson G.B. (2006). Ferroelectric thin films: Review of materials, properties, and applications. J. Appl. Phys..

[4] Ruan L., Yao X., Chang Y., Zhou L., Qin G., Zhang X. (2018). Properties and applications of the β phase poly(vinylidene fluoride). Polymers.

[5] Tian B.B., Wang J.L., Fusil S., Liu Y., Zhao X.L., Sun S., Shen H., Lin T., Sun J.L., Duan C.G. (2016). Tunnel electroresistance through organic ferroelectrics. Nat. Commun..

[6] Mathur S.C., Scheinbeim J.I., Newman B.A. (1984). Piezoelectric properties and ferroelectric hysteresis effects in uniaxially stretched nylon-11 films. J. Appl. Phys..

[7] Horiuchi S., Tokunaga Y., Giovannetti G., Picozzi S., Itoh H., Shimano R., Kumai R., Tokura Y. (2010). Above-room-temperature ferroelectricity in a single-component molecular crystal. Nature.

[8] Fu D.W., Cai H.L., Liu Y., Ye Q., Zhang W., Zhang Y., Chen X.Y., Giovannetti G., Capone M., Li J. (2013). Diisopropylammonium bromide is a high-temperature molecular ferroelectric crystal. Science.

[9] Tian B., Liu L., Yan M., Wang J., Zhao Q., Zhong N., Xiang P., Sun L., Peng H., Shen H. (2019). A robust artificial synapse based on organic ferroelectric polymer. Adv. Electron. Mater..

[10] Furukawa T. (1989). Ferroelectric properties of vinylidene fluoride copolymers. Ph. Transit..

[11] Nalwa H.S. (2002). Handbook of Thin Film Materials.

[12] Koseki Y., Aimi K., Ando S. (2012). Crystalline structure and molecular mobility of PVDF chains in PVDF/PMMA blend films analyzed by solid-state 19F MAS NMR spectroscopy. Polym. J..

[13] Nalwa H.S. (1995). Ferroelectric Polymers: Chemistry, Physics, and Applications.

[14] Wang Z.-Y., Fan H.-Q., Su K.-H., Wen Z.-Y. (2006). Structure and piezoelectric properties of poly(vinylidene fluoride) studied by density functional theory. Polymer.

[15] Wang Z.-Y., Fan H.-Q., Su K.-H., Wang X., Wen Z.-Y. (2007). Structure, phase transition and electric properties of poly(vinylidene fluoride-trifluoroethylene) copolymer studied with density functional theory. Polymer.

[16] Ortiz E., Cuán A., Badillo C., Cortés-Romero C.M., Wang Q., Noreña L. (2009). Electronic properties of poly(vinylidene fluoride): A density functional theory study. Mol. Simul..

[17] Correia H.M.G., Ramos M.M.D. (2005). Quantum modelling of poly(vinylidene fluoride). Comput. Mater. Sci..

[18] Martins P., Lopes A.C., Lanceros-Mendez S. (2014). Electroactive phases of poly(vinylidene fluoride): Determination, processing and applications. Prog. Polym. Sci..

[19] Ribeiro C., Costa C.M., Correia D.M., Nunes-Pereira J., Oliveira J., Martins P., Goncalves R., Cardoso V.F., Lanceros-Mendez S. (2018). Electroactive poly(vinylidene fluoride)-based structures for advanced applications. Nat. Protoc..

[20] Al-Jishi R., Taylor P.L. (1985). Equilibrium polarization and piezoelectric and pyroelectric coefficients in poly(vinylidene fluoride). J. Appl. Phys..

[21] Gomes J., Serrado Nunes J., Sencadas V., Lanceros-Mendez S. (2010). Influence of the β-phase content and degree of crystallinity on the piezo- and ferroelectric properties of poly(vinylidene fluoride). Smart Mater. Struct..

[22] Dawson N.M., Atencio P.M., Malloy K.J. (2017). Facile deposition of high quality ferroelectric poly(vinylidene fluoride) thin films by thermally modulated spin coating. J. Polym. Sci. Part B Polym. Phys..

[23] Zhu H., Yamamoto S., Matsui J., Miyashita T., Mitsuishi M. (2014). Ferroelectricity of poly(vinylidene fluoride) homopolymer Langmuir–Blodgett nanofilms. J. Mater. Chem. C.

[24] Furukawa T., Wen J.X., Suzuki K., Takashina Y., Date M. (1984). Piezoelectricity and pyroelectricity in vinylidene fluoride/trifluoroethylene copolymers. J. Appl. Phys..

[25] Cardoso V.F., Costa C.M., Minas G., Lanceros-Mendez S. (2012). Improving the optical and electroactive response of poly(vinylidene fluoride-trifluoroethylene) spin-coated films for sensor and actuator applications. Smart Mater. Struct..

[26] Bune A.V., Zhu C., Ducharme S., Blinov L.M., Fridkin V.M., Palto S.P., Petukhova N.G., Yudin S.G. (1999). Piezoelectric and pyroelectric properties of ferroelectric Langmuir–Blodgett polymer films. J. Appl. Phys..

[27] Blinov L.M., Fridkin V.M., Palto S.P., Bune A.V., Dowben P.A., Ducharme S. (2000). Two-dimensional ferroelectrics. Phys. Uspekhi.

[28] Li Z., Wang Y., Cheng Z.Y. (2006). Electromechanical properties of poly(vinylidene-fluoride-chlorotrifluoroethylene) copolymer. Appl. Phys. Lett..

[29] Kim R.H., Kang S.J., Bae I., Choi Y.S., Park Y.J., Park C. (2012). Thin ferroelectric poly(vinylidene fluoride-chlorotrifluoro ethylene) films for thermal history independent non-volatile polymer memory. Organ. Electron..

[30] Horiuchi S., Tokura Y. (2008). Organic ferroelectrics. Nat. Mater..

[31] Panda P.K. (2009). Review: Environmental friendly lead-free piezoelectric materials. J. Mater. Sci..

[32] Warlimont H., Martienssen W. (2018). Springer Handbook of Materials Data.

[33] Smith R.T., Welsh F.S. (1971). Temperature dependence of the elastic, piezoelectric, and dielectric constants of lithium tantalate and lithium niobate. J. Appl. Phys..

[34] Park M.H., Lee Y.H., Kim H.J., Kim Y.J., Moon T., Kim K.D., Muller J., Kersch A., Schroeder U., Mikolajick T. (2015). Ferroelectricity and antiferroelectricity of doped thin HfO2-based films. Adv. Mater..

[35] Benz M., Euler W.B., Gregory O.J. (2002). The role of solution phase water on the deposition of thin films of poly(vinylidene fluoride). Macromolecules.

[36] Dmitriev I.Y., Lavrentyev V.K., Elyashevich G.K. (2006). Polymorphic transformations in poly(vinylidene fluoride) films during orientation. Polym. Sci. Series A.

[37] Kang S.J., Park Y.J., Sung J., Jo P.S., Park C., Kim K.J., Cho B.O. (2008). Spin cast ferroelectric beta poly(vinylidene fluoride) thin films via rapid thermal annealing. Appl. Phys. Lett..

[38] Ramasundaram S., Yoon S., Kim K.J., Lee J.S. (2008). Direct preparation of nanoscale thin films of poly(vinylidene fluoride) containing β-crystalline phase by heat-controlled spin coating. Macromol. Chem. Phys..

[39] California A., Cardoso V.F., Costa C.M., Sencadas V., Botelho G., Gomez-Ribelles J.L., Lanceros-Mendez S. (2011). Tailoring porous structure of ferroelectric poly(vinylidene fluoride-trifluoroethylene) by controlling solvent/polymer ratio and solvent evaporation rate. Eur. Polym. J..

[40] Cardoso V.F., Minas G., Costa C.M., Tavares C.J., Lanceros-Mendez S. (2011). Micro and nanofilms of poly(vinylidene fluoride) with controlled thickness, morphology and electroactive crystalline phase for sensor and actuator applications. Smart Mater. Struct..

[41] Cardoso V.F., Minas G., Lanceros-Mendez S. (2013). Multi layer spin-coating deposition of poly(vinylidene fluoride) films for controlling thickness and piezoelectric response. Sens. Actuators A Phys..

[42] Nishiyama T., Sumihara T., Sato E., Horibe H. (2016). Effect of solvents on the crystal formation of poly(vinylidene fluoride) film prepared by a spin-coating process. Polym. J..

[43] Mahale B., Bodas D., Gangal S.A. (2017). Study of β-phase development in spin-coated PVDF thick films. Bull. Mater. Sci..

[44] Cardoso V.F., Correia D.M., Ribeiro C., Fernandes M.M., Lanceros-Mendez S. (2018). Fluorinated Polymers as Smart Materials for Advanced Biomedical Applications. Polymers.

[45] Cardoso V.F., Marques-Almeida T., Rodrigues-Marinho T., Minas G., Rebouta L., Lanceros-Mendez S. (2018). Layer-by-layer fabrication of highly transparent polymer based piezoelectric transducers. Mater. Res. Express.

[46] Patro T.U., Mhalgi M.V., Khakhar D.V., Misra A. (2008). Studies on poly(vinylidene fluoride)–clay nanocomposites: Effect of different clay modifiers. Polymer.

[47] Thakur P., Kool A., Bagchi B., Hoque N.A., Das S., Nandy P. (2015). The role of cerium(iii)/yttrium(iii) nitrate hexahydrate salts on electroactive β phase nucleation and dielectric properties of poly(vinylidene fluoride) thin films. RSC Adv..

[48] Mandal D., Kim K.J., Lee J.S. (2012). Simple synthesis of palladium nanoparticles, β-phase formation, and the control of chain and dipole orientations in palladium-doped poly(vinylidene fluoride) thin films. Langmuir.

[49] Wang W., Zhang S., Srisombat L.-o., Lee T.R., Advincula R.C. (2011). Gold-Nanoparticle- and Gold-Nanoshell-Induced Polymorphism in Poly(vinylidene fluoride). Macromol. Mater. Eng..

[50] Ghosh S.K., Alam M.M., Mandal D. (2014). The in situ formation of platinum nanoparticles and their catalytic role in electroactive phase formation in poly(vinylidene fluoride): A simple preparation of multifunctional poly(vinylidene fluoride) films doped with platinum nanoparticles. RSC Adv..

[51] Li M., Wondergem H.J., Spijkman M.J., Asadi K., Katsouras I., Blom P.W., de Leeuw D.M. (2013). Revisiting the δ-phase of poly(vinylidene fluoride) for solution-processed ferroelectric thin films. Nat. Mater..

[52] Müller K., Mandal D., Henkel K., Paloumpa I., Schmeisser D. (2008). Ferroelectric properties of spin-coated ultrathin (down to 10 nm) copolymer films. Appl. Phys. Lett..

[53] Nakajima T., Takahashi Y., Okamura S., Furukawa T. (2009). Nanosecond switching characteristics of ferroelectric ultrathin vinylidene fluoride/trifluoroethylene copolymer films under extremely high electric field. Jpn. J. Appl. Phys..

[54] Kim Y., Hong S., Oh S., Choi Y.-Y., Choi H., No K. (2015). The effects of an alkaline treatment on the ferroelectric properties of poly(vinylidene fluoride trifluoroethylene) films. Electron. Mater. Lett..

[55] Bune A.V., Fridkin V.M., Ducharme S., Blinov L.M., Palto S.P., Sorokin A.V., Yudin S.G., Zlatkin A. (1998). Two-dimensional ferroelectric films. Nature.

[56] Cui C., Xue F., Hu W.-J., Li L.-J. (2018). Two-dimensional materials with piezoelectric and ferroelectric functionalities. Npj 2D Mater. Appl..

[57] Palto S., Blinov L., Bune A., Dubovik E., Fridkin V., Petukhova N., Verkhovskaya K., Yudin S. (1995). Ferroelectric Langmuir-Blodgett films. Ferroelectr. Lett. Sect..

[58] Blinov L.M., Fridkin V.M., Palto S.P., Sorokin A.V., Yudin S.G. (1996). Ferroelectric polymer Langmuir films. Thin Solid Films.

[59] Zhu H., Yamamoto S., Matsui J., Miyashita T., Mitsuishi M. (2016). Highly oriented poly(vinylidene fluoride-co-trifluoroethylene) ultrathin films with improved ferroelectricity. RSC Adv..

[60] Bai M., Ducharme S. (2004). Ferroelectric nanomesa formation from polymer Langmuir–Blodgett films. Appl. Phys. Lett..

[61] Bai M., Poulsen M., Ducharme S. (2006). Effects of annealing conditions on ferroelectric nanomesa self-assembly. J. Phys. Condens. Matter.

[62] Li J., Luo Y., Bai M., Ducharme S. (2005). Nanomesa and nanowell formation in Langmuir–Blodgett polyvinylidene fluoride trifluoroethylene copolymer films. Appl. Phys. Lett..

[63] Li J., Luo Y., Bai M., Ducharme S. (2006). A continuum model on the nanomesa and nanowell formation in Langmuir–Blodgett ferroelectric polymeric films. J. Mech. Phys. Solids.

[64] Li J., Luo Y., Bai M., Ducharme S. Nanomesa and nanowell formations in Langmuir–Blodgett polyvinylidene fluoride trifluoroethelyne copolymer films. Proceedings of the Smart Structures and Materials 2006: Electroactive Polymer Actuators and Devices (EAPAD).

[65] Cai X., Lei T., Sun D., Lin L. (2017). A critical analysis of the α, β and γ phases in poly(vinylidene fluoride) using FTIR. RSC Adv..

[66] Gregorio R., Ueno E.M. (1999). Effect of crystalline phase, orientation and temperature on the dielectric properties of poly (vinylidene fluoride) (PVDF). J. Mater. Sci..

[67] Gregorio R., Cestari M. (1994). Effect of Crystallization Temperature on the Crystalline Phase Content and Morphology of Poly (vinylidene Fluoride). J. Polym. Sci. Part B Polym. Phys..

[68] Gregorio R., Capitao R.C. (2000). Morphology and phase transition of high melt temperature crystallized poly(vinylidene fluoride). J. Mater. Sci..

[69] Gregorio R. (2006). Determination of the α, β, and γ crystalline phases of poly(vinylidene fluoride) films prepared at different conditions. J. Appl. Polym. Sci..

[70] Boccaccio T., Bottino A., Capannelli G., Piaggio P. (2002). Characterization of PVDF membranes by vibrational spectroscopy. J. Membr. Sci..

[71] Imamura R., Silva A.B., Gregorio R. (2008). γ→β Phase transformation induced in poly(vinylidene fluoride) by stretching. J. Appl. Polym. Sci..

[72] Benz M., Euler W.B. (2003). Determination of the crystalline phases of poly(vinylidene fluoride) under different preparation conditions using differential scanning calorimetry and infrared spectroscopy. J. Appl. Polym. Sci..

[73] Milani A., Castiglioni C., Radice S. (2015). Joint experimental and computational investigation of the structural and spectroscopic properties of poly(vinylidene fluoride) polymorphs. J. Phys. Chem. B.

[74] Li J.C., Wang C.L., Zhong W.L., Zhang P.L., Wang Q.H., Webb J.F. (2002). Vibrational mode analysis of β-phase poly(vinylidene fluoride). Appl. Phys. Lett..

[75] Correia H.M.G., Ramos M.M.D. (2011). What can we learn from vibrational analysis calculations of defective polymer chains?. Ferroelectrics.

[76] Ramer N.J., Marrone T., Stiso K.A. (2006). Structure and vibrational frequency determination for α-poly(vinylidene fluoride) using density-functional theory. Polymer.

[77] Buckley J., Cebe P., Cherdack D., Crawford J., Ince B.S., Jenkins M., Pan J., Reveley M., Washington N., Wolchover N. (2006). Nanocomposites of poly(vinylidene fluoride) with organically modified silicate. Polymer.

[78] Esterly D.M., Love B.J. (2004). Phase transformation to β-Poly(vinylidene fluoride) by milling. J. Polym. Sci. Part B Polym. Phys..

[79] Davis G.T., McKinney J.E., Broadhurst M.G., Roth S.C. (1978). Electric-field-induced phase changes in poly(vinylidene fluoride). J. Appl. Phys..

[80] Hasegawa R., Takahashi Y., Chatani Y., Adokoro H.T. (1972). Crystal Structures of Three Crystalline Forms of Poly(vinylidene fluoride). Polym. J..

[81] Okada D., Kaneko H., Kato K., Furumi S., Takeguchi M., Yamamoto Y. (2015). Colloidal Crystallization and Ionic Liquid Induced Partial β-Phase Transformation of Poly(vinylidene fluoride) Nanoparticles. Macromolecules.

[82] Hong S. (2004). Nanoscale Phenomena in Ferroelectric Films.

[83] Naber R.C.G., Blom P.W.M., Leeuw D.M.D. (2006). Comment on ‘Extrinsic versus intrinsic ferroelectric switching: Experimental investigations using ultra-thin PVDF Langmuir–Blodgett films’. J. Phys. D Appl. Phys..

[84] Ducharme S., Fridkin V.M., Bune A.V., Palto S.P., Blinov L.M., Petukhova N.N., Yudin S.G. (2000). Intrinsic ferroelectric coercive field. Phys. Rev. Lett..

[85] Fridkin V., Ducharme S. (2014). The Ferroelectricity at the Nanoscale. Ferroelectrics.

[86] Tagantsev A.K., Stolichnov I., Setter N., Cross J.S., Tsukada M. (2002). Non-Kolmogorov-Avrami switching kinetics in ferroelectric thin films. Phys. Rev. B.

[87] Jo J.Y., Han H.S., Yoon J.G., Song T.K., Kim S.H., Noh T.W. (2007). Domain switching kinetics in disordered ferroelectric thin films. Phys. Rev. Lett..

[88] Mao D., Mejia I., Stiegler H., Gnade B.E., Quevedo-Lopez M.A. (2010). Polarization behavior of poly(vinylidene fluoride-trifluoroethylene) copolymer ferroelectric thin film capacitors for nonvolatile memory application in flexible electronics. J. Appl. Phys..

[89] So Y.W., Kim D.J., Noh T.W., Yoon J.-G., Song T.K. (2005). Polarization switching kinetics of epitaxial Pb(Zr_0.4_Ti_0.6_)O_3_ thin films. Appl. Phys. Lett..

[90] Hu W.J., Juo D.M., You L., Wang J., Chen Y.C., Chu Y.H., Wu T. (2014). Universal ferroelectric switching dynamics of vinylidene fluoride-trifluoroethylene copolymer films. Sci. Rep..

[91] Zhukov S., Genenko Y.A., Hirsch O., Glaum J., Granzow T., von Seggern H. (2010). Dynamics of polarization reversal in virgin and fatigued ferroelectric ceramics by inhomogeneous field mechanism. Phys. Rev. B.

[92] Schütrumpf J., Zhukov S., Genenko Y.A., von Seggern H. (2012). Polarization switching dynamics by inhomogeneous field mechanism in ferroelectric polymers. J. Phys. D Appl. Phys..

[93] Mai M., Ke S., Lin P., Zeng X. (2015). Ferroelectric Polymer Thin Films for Organic Electronics. J. Nanomater..

[94] Kalinin S.V., Gruverman A. (2010). Scanning Probe Microscopy of Functional Materials: Nanoscale Imaging and Spectroscopy.

[95] Alexe M., Gruverman A. (2004). Nanoscale Characterisation of Ferroelectric Materials: Scanning Probe Microscopy Approach.

[96] Güthner P., Dransfeld K. (1992). Local poling of ferroelectric polymers by scanning force microscopy. Appl. Phys. Lett..

[97] Gruverman A., Alexe M., Meier D. (2019). Piezoresponse force microscopy and nanoferroic phenomena. Nat. Commun..

[98] Bonnell D.A., Kalinin S.V. (2013). Scanning Probe Microscopy for Energy Research.

[99] Foster A., Hofer W. (2006). Scanning Probe Microscopy: Atomic Scale Engineering by Forces and Currents.

[100] Kalinin S., Gruverman A. (2007). Scanning Probe Microscopy: Electrical and Electromechanical Phenomena at the Nanoscale.

[101] Voigtländer B. (2015). Scanning Probe Microscopy: Atomic Force Microscopy and Scanning Tunneling Microscopy.

[102] Merz W.J. (1954). Domain formation and domain wall motions in ferroelectric BaTiO_3_ single crystals. Phys. Rev..

[103] Merz W.J. (1956). Switching time in ferroelectric BaTiO_3_ and its dependence on crystal thickness. J. Appl. Phys..

[104] Little E.A. (1955). Dynamic Behavior of Domain Walls in Barium Titanate. Phys. Rev..

[105] Miller R.C. (1958). Some experiments on the motion of 180° domain walls in BaTiO_3_. Phys. Rev..

[106] Miller R.C., Weinreich G. (1960). Mechanism for the sidewise motion of 180° domain walls in barium titanate. Phys. Rev..

[107] Fatuzzo E. (1962). Theoretical Considerations on the Switching Transient in Ferroelectrics. Phys. Rev..

[108] Kolmogorov A.N. (1937). A statistical theory for the recrystallization of metals. Izv. Akad. Nauk USSR Serie Math..

[109] Orihara H., Hashimoto S., Ishibashi Y. (1994). A theory of D-E hysteresis loop based on the Avrami model. J. Phys. Soc. Jpn..

[110] Kliem H., Tadros-Morgane R. (2005). Extrinsic versus intrinsic ferroelectric switching: Experimental investigations using ultra-thin PVDF Langmuir–Blodgett films. J. Phys. D Appl. Phys..

[111] Paramonova E.V., Filippov S.V., Gevorkyan V.E., Avakyan L.A., Meng X.J., Tian B.B., Wang J.L., Bystrov V.S. (2017). Polarization switching in ultrathin polyvinylidene fluoride homopolymer ferroelectric films. Ferroelectrics.

[112] Li W., Alexe M. (2007). Investigation on switching kinetics in epitaxial Pb(Zr_0.2_Ti_0.8_)O_3_ ferroelectric thin films: Role of the 90° domain walls. Appl. Phys. Lett..

[113] Tagantsev A.K., Cross L.E., Fousek J. (2010). Domains in Ferroic Crystals and Thin Films.

[114] Chen I.W., Wang Y. (1999). Activation field and fatigue of (Pb, La)(Zr, Ti)O_3_ thin films. Appl. Phys. Lett..

[115] Jung D.J., Dawber M., Scott J.F., Sinnamon L.J., Gregg J.M. (2010). Switching dynamics in ferroelectric thin films: An experimental survey. Integr. Ferroelectr..

[116] Tian B.B., Chen L.F., Liu Y., Bai X.F., Wang J.L., Sun S., Yuan G.L., Sun J.L., Dkhil B., Meng X.J. (2015). Homogeneous switching mechanism in pure polyvinylidene fluoride ultrathin films. Phys. Rev. B.

[117] Fridkin V., Ducharme S. (2014). Ferroelectricity at the Nanoscale: Basics and Applications.

[118] Highland M.J., Fister T.T., Richard M.I., Fong D.D., Fuoss P.H., Thompson C., Eastman J.A., Streiffer S.K., Stephenson G.B. (2010). Polarization switching without domain formation at the intrinsic coercive field in ultrathin ferroelectric PbTiO_3_. Phys. Rev. Lett..

[119] Vizdrik G., Ducharme S., Fridkin V.M., Yudin S.G. (2003). Kinetics of ferroelectric switching in ultrathin films. Phys. Rev. B.

[120] Fridkin V., Ievlev A., Verkhovskaya K., Vizdrik G., Yudin S., Ducharme S. (2005). Switching in one monolayer of the ferroelectric polymer. Ferroelectrics.

[121] Gaynutdinov R., Yudin S., Ducharme S., Fridkin V. (2012). Homogeneous switching in ultrathin ferroelectric films. J. Phys. Condens. Matter.

[122] Gaynutdinov R.V., Mitko S., Yudin S.G., Fridkin V.M., Ducharme S. (2011). Polarization switching at the nanoscale in ferroelectric copolymer thin films. Appl. Phys. Lett..

[123] Ducharme S., Fridkin V., Gaynutdinov R., Minnekaev M., Tolstikhina A., Zenkevich A. (2012). Homogeneous switching in ultrathin ferroelectric BaTiO_3_ films. arXiv.

[124] Chen X., Han X., Shen Q.-D. (2017). PVDF-based ferroelectric polymers in modern flexible electronics. Adv. Electron. Mater..

[125] Waser R., Dittmann R., Staikov G., Szot K. (2009). Redox-based resistive switching memories—Nanoionic mechanisms, prospects, and challenges. Adv. Mater..

[126] Ouyang J. (2016). Emerging Resistive Switching Memories.

[127] Waser R., Dittmann R., Menzel S., Noll T. (2019). Introduction to new memory paradigms: Memristive phenomena and neuromorphic applications. Faraday Discuss.

[128] Chen A. (2016). A review of emerging non-volatile memory (NVM) technologies and applications. Solid State Electron..

[129] Scott J.F. (2000). Ferroelectric Memories.

[130] Park Y.J., Bae I.-S., Kang S.J., Chang J., Park C. (2010). Control of thin ferroelectric polymer films for non-volatile memory applications. IEEE Trans. Dielectr. Electr. Insul..

[131] Reece T.J., Ducharme S., Sorokin A.V., Poulsen M. (2003). Nonvolatile memory element based on a ferroelectric polymer Langmuir–Blodgett film. Appl. Phys. Lett..

[132] Lim S.H., Rastogi A.C., Desu S.B. (2004). Electrical properties of metal-ferroelectric-insulator-semiconductor structures based on ferroelectric polyvinylidene fluoride copolymer film gate for nonvolatile random access memory application. J. Appl. Phys..

[133] Gerber A., Kohlstedt H., Fitsilis M., Waser R., Reece T.J., Ducharme S., Rije E. (2006). Low-voltage operation of metal-ferroelectric-insulator-semiconductor diodes incorporating a ferroelectric polyvinylidene fluoride copolymer Langmuir-Blodgett film. J. Appl. Phys..

[134] Takahashi M., Kodama K., Nakaiso T., Noda M., Okuyama M. (2006). Effect of leakage current through ferroelectric and insulator on retention characteristics of metal-ferroelectric-insulator-semiconductor structure. Integr. Ferroelectr..

[135] Fujisaki S., Ishiwara H., Fujisaki Y. (2007). Low-voltage operation of ferroelectric poly(vinylidene fluoride-trifluoroethylene) copolymer capacitors and metal-ferroelectric-insulator-semiconductor diodes. Appl. Phys. Lett..

[136] Fujisaki S., Ishiwara H., Fujisaki Y. (2008). Organic ferroelectric diodes with long retention characteristics suitable for non-volatile memory applications. Appl. Phys. Express.

[137] Furukawa T., Kanai S., Okada A., Takahashi Y., Yamamoto R. (2009). Ferroelectric switching dynamics in VDF-TrFE copolymer thin films spin coated on Si substrate. J. Appl. Phys..

[138] Henkel K., Lazareva I., Mandal D., Paloumpa I., Müller K., Koval Y., Müller P., Schmeißer D. (2009). Electrical investigations on metal/ferroelectric/insulator/semiconductor structures using poly[vinylidene fluoride trifluoroethylene] as ferroelectric layer for organic nonvolatile memory applications. J. Vac. Sci. Technol. B Microelectron. Nanometer Struct..

[139] Lu X., Yoon J.-W., Ishiwara H. (2009). Low-voltage operation and excellent data retention characteristics of metalferroelectric-insulator-Si devices based on organic ferroelectric films. J. Appl. Phys..

[140] Park Y.J., Kang S.J., Shin Y., Kim R.H., Bae I., Park C. (2011). Non-volatile memory characteristics of epitaxially grown PVDF-TrFE thin films and their printed micropattern application. Curr. Appl. Phys..

[141] Miller S.L., McWhorter P.J. (1992). Physics of the ferroelectric nonvolatile memory field effect transistor. J. Appl. Phys..

[142] Lue H.-T., Wu C.-J., Tseng T.-Y. (2002). Device modeling of ferroelectric memory field-effect transistor (FeMFET). IEEE Trans. Electron. Devices.

[143] Tokumitsu E., Fujii G., Ishiwara H. (2000). Electrical properties of metal-ferroelectric-insulator-semiconductor (MFIS)- and metal-ferroelectric-metal-insulator-semiconductor (MFMIS)-FETs using ferroelectric SrBi_2_Ta_2_O_9_ film and SrTa_2_O_6_/SiON buffer layer. Jpn. J. Appl. Phys..

[144] Naber R.C.G., Tanase C., Blom P.W.M., Gelinck G.H., Marsman A.W., Touwslager F.J., Setayesh S., de Leeuw D.M. (2005). High-performance solution-processed polymer ferroelectric field-effect transistors. Nat. Mater..

[145] Gerber A., Fitsilis M., Waser R., Reece T.J., Rije E., Ducharme S., Kohlstedt H. (2010). Ferroelectric field effect transistors using very thin ferroelectric polyvinylidene fluoride copolymer films as gate dielectrics. J. Appl. Phys..

[146] Yoon S.-M., Yang S., Byun C.-W., Jung S.-W., Ryu M.-K., Park S.-H.K., Kim B., Oh H., Hwang C.-S., Yu B.-G. (2011). Nonvolatile memory thin-film transistors using an organic ferroelectric gate insulator and an oxide semiconducting channel. Semicond. Sci. Technol..

[147] Miyata Y., Yoshimura T., Ashida A., Fujimura N. (2016). Low-voltage operation of Si-based ferroelectric field effect transistors using organic ferroelectrics, poly(vinylidene fluoride–trifluoroethylene), as a gate dielectric. Jpn. J. Appl. Phys..

[148] Asai H., Fukuda K., Hattori J., Koike H., Miyata N., Takahashi M., Sakai S. (2017). Compact model of ferroelectric-gate field-effect transistor for circuit simulation based on multidomain Landau–Kalathnikov theory. Jpn. J. Appl. Phys..

[149] Esaki L., Laibowitz R.B., Stiles P.J. (1971). Polar switch. IBM Tech. Discl. Bull..

[150] Garcia V., Fusil S., Bouzehouane K., Enouz-Vedrenne S., Mathur N.D., Barthelemy A., Bibes M. (2009). Giant tunnel electroresistance for non-destructive readout of ferroelectric states. Nature.

[151] Gruverman A., Wu D., Lu H., Wang Y., Jang H.W., Folkman C.M., Zhuravlev M.Y., Felker D., Rzchowski M., Eom C.-B. (2009). Tunneling electroresistance effect in ferroelectric tunnel junctions at the nanoscale. Nano Lett..

[152] Zenkevich A., Minnekaev M., Matveyev Y., Lebedinskii Y., Bulakh K., Chouprik A., Baturin A., Maksimova K., Thiess S., Drube W. (2013). Electronic band alignment and electron transport in Cr/BaTiO_3_/Pt ferroelectric tunnel junctions. Appl. Phys. Lett..

[153] Kohlstedt H., Pertsev N.A., Rodríguez Contreras J., Waser R. (2005). Theoretical current-voltage characteristics of ferroelectric tunnel junctions. Phys. Rev. B.

[154] Tsymbal E.Y., Kohlstedt H. (2006). Tunneling across a ferroelectric. Science.

[155] Zhuravlev M.Y., Sabirianov R.F., Jaswal S.S., Tsymbal E.Y. (2005). Giant electroresistance in ferroelectric tunnel junctions. Phys. Rev. Lett..

[156] Garcia V., Bibes M. (2014). Ferroelectric tunnel junctions for information storage and processing. Nat. Commun..

[157] Liang S., Yu Z., Devaux X., Ferri A., Huang W., Yang H., Desfeux R., Li X., Migot S., Chaudhuri D. (2018). Quenching of spin polarization switching in organic multiferroic tunnel junctions by ferroelectric “ailing-channel” in organic barrier. ACS Appl. Mater. Interfaces.

[158] Chanthbouala A., Crassous A., Garcia V., Bouzehouane K., Fusil S., Moya X., Allibe J., Dlubak B., Grollier J., Xavier S. (2011). Solid-state memories based on ferroelectric tunnel junctions. Nat. Nanotechnol..

[159] Pantel D., Lu H., Goetze S., Werner P., Jik Kim D., Gruverman A., Hesse D., Alexe M. (2012). Tunnel electroresistance in junctions with ultrathin ferroelectric Pb(Zr_0.2_Ti_0.8_)O_3_ barriers. Appl. Phys. Lett..

[160] Wen Z., Li C., Wu D., Li A., Ming N. (2013). Ferroelectric-field-effect-enhanced electroresistance in metal/ferroelectric/semiconductor tunnel junctions. Nat. Mater..

[161] Xi Z., Ruan J., Li C., Zheng C., Wen Z., Dai J., Li A., Wu D. (2017). Giant tunnelling electroresistance in metal/ferroelectric/semiconductor tunnel junctions by engineering the Schottky barrier. Nat. Commun..

[162] Qu H., Yao W., Garcia T., Zhang J., Sorokin A.V., Ducharme S., Dowben P.A., Fridkin V.M. (2003). Nanoscale polarization manipulation and conductance switching in ultrathin films of a ferroelectric copolymer. Appl. Phys. Lett..

[163] Kusuma D.Y., Lee P.S. (2012). Ferroelectric tunnel junction memory devices made from monolayers of vinylidene fluoride oligomers. Adv. Mater..

[164] Moodera J.S., Kinder L.R., Wong T.M., Meservey R. (1995). Large magnetoresistance at room temperature in ferromagnetic thin film tunnel junctions. Phys. Rev. Lett..

[165] Parkin S.S., Kaiser C., Panchula A., Rice P.M., Hughes B., Samant M., Yang S.H. (2004). Giant tunnelling magnetoresistance at room temperature with MgO (100) tunnel barriers. Nat. Mater..

[166] Gajek M., Bibes M., Fusil S., Bouzehouane K., Fontcuberta J., Barthelemy A., Fert A. (2007). Tunnel junctions with multiferroic barriers. Nat. Mater..

[167] Pantel D., Goetze S., Hesse D., Alexe M. (2012). Reversible electrical switching of spin polarization in multiferroic tunnel junctions. Nat. Mater..

[168] Yin Y.W., Raju M., Hu W.J., Burton J.D., Kim Y.M., Borisevich A.Y., Pennycook S.J., Yang S.M., Noh T.W., Gruverman A. (2015). Multiferroic tunnel junctions and ferroelectric control of magnetic state at interface (invited). J. Appl. Phys..

[169] Fiebig M., Lottermoser T., Meier D., Trassin M. (2016). The evolution of multiferroics. Nat. Rev. Mater..

[170] Spaldin N.A., Ramesh R. (2019). Advances in magnetoelectric multiferroics. Nat. Mater..

[171] Lopez-Encarnacion J.M., Burton J.D., Tsymbal E.Y., Velev J.P. (2011). Organic multiferroic tunnel junctions with ferroelectric poly(vinylidene fluoride) barriers. Nano Lett..

[172] Liang S., Yang H., Yang H., Tao B., Djeffal A., Chshiev M., Huang W., Li X., Ferri A., Desfeux R. (2016). Ferroelectric control of organic/ferromagnetic spinterface. Adv. Mater..

[173] Yang J.J., Strukov D.B., Stewart D.R. (2013). Memristive devices for computing. Nat. Nanotechnol..

[174] Chua L.O. (1971). Memristor—The missing circuit element. IEEE Trans. Circuit Theory.

[175] Chua L.O., Kang S.M. (1976). Memristive devices and systems. Proc. IEEE.

[176] Strukov D.B., Snider G.S., Stewart D.R., Williams R.S. (2008). The missing memristor found. Nature.

[177] Jo S.H., Chang T., Ebong I., Bhadviya B.B., Mazumder P., Lu W. (2010). Nanoscale memristor device as synapse in neuromorphic systems. Nano Lett..

[178] Ohno T., Hasegawa T., Tsuruoka T., Terabe K., Gimzewski J.K., Aono M. (2011). Short-term plasticity and long-term potentiation mimicked in single inorganic synapses. Nat. Mater..

[179] Yu S., Gao B., Fang Z., Yu H., Kang J., Wong H.S. (2013). A low energy oxide-based electronic synaptic device for neuromorphic visual systems with tolerance to device variation. Adv. Mater..

[180] Prezioso M., Merrikh-Bayat F., Hoskins B.D., Adam G.C., Likharev K.K., Strukov D.B. (2015). Training and operation of an integrated neuromorphic network based on metal-oxide memristors. Nature.

[181] Serb A., Bill J., Khiat A., Berdan R., Legenstein R., Prodromakis T. (2016). Unsupervised learning in probabilistic neural networks with multi-state metal-oxide memristive synapses. Nat. Commun..

[182] Yao P., Wu H., Gao B., Eryilmaz S.B., Huang X., Zhang W., Zhang Q., Deng N., Shi L., Wong H.P. (2017). Face classification using electronic synapses. Nat. Commun..

[183] Wang Z., Joshi S., Savel’ev S., Song W., Midya R., Li Y., Rao M., Yan P., Asapu S., Zhuo Y. (2018). Fully memristive neural networks for pattern classification with unsupervised learning. Nat. Electron..

[184] Borghetti J., Snider G.S., Kuekes P.J., Yang J.J., Stewart D.R., Williams R.S. (2010). Memristive switches enable stateful logic operations via material implication. Nature.

[185] Linn E., Rosezin R., Tappertzhofen S., Bottger U., Waser R. (2012). Beyond von Neumann—Logic operations in passive crossbar arrays alongside memory operations. Nanotechnology.

[186] Zidan M.A., Strachan J.P., Lu W.D. (2018). The future of electronics based on memristive systems. Nat. Electron..

[187] Li C., Hu M., Li Y., Jiang H., Ge N., Montgomery E., Zhang J., Song W., Dávila N., Graves C.E. (2017). Analogue signal and image processing with large memristor crossbars. Nat. Electron..

[188] Bi G.-Q., Poo M.-M. (1998). Synaptic modifications in cultured hippocampal neurons: Dependence on spike timing, synaptic strength, and postsynaptic cell type. J. Neurosci..

[189] van de Burgt Y., Melianas A., Keene S.T., Malliaras G., Salleo A. (2018). Organic electronics for neuromorphic computing. Nat. Electron..

[190] Yu S., Li Z., Chen P.-Y., Wu H., Gao B., Wang D., Wu W., Qian H. Binary Neural Network with 16 Mb RRAM Macro Chip for Classification and Online Training. Proceedings of the IEEE International Electron Devices Meeting (IEDM).

[191] Yu S., Yu S. (2017). Neuro-Inspired Computing Using Resistive Synaptic Devices.

[192] Chanthbouala A., Garcia V., Cherifi R.O., Bouzehouane K., Fusil S., Moya X., Xavier S., Yamada H., Deranlot C., Mathur N.D. (2012). A ferroelectric memristor. Nat. Mater..

[193] Kim D.J., Lu H., Ryu S., Bark C.W., Eom C.B., Tsymbal E.Y., Gruverman A. (2012). Ferroelectric tunnel memristor. Nano Lett..

[194] Hambe M., Petraru A., Pertsev N.A., Munroe P., Nagarajan V., Kohlstedt H. (2010). Crossing an interface: Ferroelectric control of tunnel currents in magnetic complex oxide heterostructures. Adv. Funct. Mater..

[195] Tian B.B., Liu Y., Chen L.F., Wang J.L., Sun S., Shen H., Sun J.L., Yuan G.L., Fusil S., Garcia V. (2015). Space-charge effect on electroresistance in metalferroelectric-metal capacitors. Sci. Rep..

[196] Boyn S., Grollier J., Lecerf G., Xu B., Locatelli N., Fusil S., Girod S., Carretero C., Garcia K., Xavier S. (2017). Learning through ferroelectric domain dynamics in solid-state synapses. Nat. Commun..

[197] Chen L., Wang T.Y., Dai Y.W., Cha M.Y., Zhu H., Sun Q.Q., Ding S.J., Zhou P., Chua L., Zhang D.W. (2018). Ultra-low power Hf_0.5_Zr_0.5_O_2_ based ferroelectric tunnel junction synapses for hardware neural network applications. Nanoscale.

[198] Majumdar S., Tan H., Qin Q.H., van Dijken S. (2019). Energy-efficient organic ferroelectric tunnel junction memristors for neuromorphic computing. Adv. Electron. Mater..

[199] Chen D., Sharma T., Chen Y., Fu X., Zhang J.X.J. Gold nanoparticles doped flexible PVDF-TrFE energy harvester. Proceedings of the 8th Annual IEEE International Conference on Nano/Micro Engineered and Molecular Systems.

[200] Toprak A., Tigli O. (2015). MEMS scale PVDF-TrFE-based piezoelectric energy harvesters. J. Microelectromech. Syst..

[201] Pi Z., Zhang J., Wen C., Zhang Z.-b., Wu D. (2014). Flexible piezoelectric nanogenerator made of poly(vinylidenefluoride-co-trifluoroethylene) (PVDF-TrFE) thin film. Nano Energy.

[202] Hu P., Yan L., Zhao C., Zhang Y., Niu J. (2018). Double-layer structured PVDF nanocomposite film designed for flexible nanogenerator exhibiting enhanced piezoelectric output and mechanical property. Compos. Sci. Technol..

[203] Cui N., Gu L., Liu J., Bai S., Qiu J., Fu J., Kou X., Liu H., Qin Y., Wang Z.L. (2015). High performance sound driven triboelectric nanogenerator for harvesting noise energy. Nano Energy.

[204] Chen X.-Z., Chen X., Guo X., Cui Y.-S., Shen Q.-D., Ge H.-X. (2014). Ordered arrays of a defect-modified ferroelectric polymer for non-volatile memory with minimized energy consumption. Nanoscale.

[205] Chen X.-Z., Li Z.-W., Cheng Z.-X., Zhang J.-Z., Shen Q.-D., Ge H.-X., Li H.-T. (2011). Greatly enhanced energy density and patterned films induced by photo crosslinking of poly(vinylidene fluoridechlorotrifluoroethylene). Macromol. Rapid Commun..

[206] Chen X.-Z., Li Q., Chen X., Guo X., Ge H.-X., Liu Y., Shen Q.-D. (2013). Nano-imprinted ferroelectric polymer nanodot arrays for high density data storage. Adv. Funct. Mater..

[207] Wang Z.L., Song J. (2006). Piezoelectric nanogenerators based on zinc oxide nanowire arrays. Science.

[208] Toprak A., Tigli O. (2017). Comprehensive characterization of PVDF-TrFE thin films for microelectromechanical system applications. J. Mater. Sci. Mater. Electron..

[209] Sharma T., Je S.-S., Gill B., Zhang J.X.J. (2012). Patterning piezoelectric thin film PVDF–TrFE based pressure sensor for catheter application. Sens. Actuators A Phys..

[210] Dahiya R.S., Valle M., Metta G., Lorenzelli L. Bio-inspired tactile sensing arrays. Proceedings of the Bioengineered and Bioinspired Systems IV.

[211] Dahiya R.S., Valle M., Metta G., Lorenzelli L., Adami A. Design and fabrication of POSFET devices for tactile sensing. Proceedings of the Transducers 2009–2009 International Solid-State Sensors, Actuators and Microsystems Conference.

[212] Jel S.S., Sharma T., Lee Y., Gill B., Zhang J.X. A thin-film piezoelectric PVDF-TrFE based implantable pressure sensor using lithographic patterning. Proceedings of the IEEE International Conference on Micro Electro Mechanical Systems (MEMS).

[213] Viola F.A., Spanu A., Ricci P.C., Bonfiglio A., Cosseddu P. (2018). Ultrathin, flexible and multimodal tactile sensors based on organic field-effect transistors. Sci. Rep..

[214] Shin K.-Y., Lee J.S., Jang J. (2016). Highly sensitive, wearable and wireless pressure sensor using free-standing ZnO nanoneedle/PVDF hybrid thin film for heart rate monitoring. Nano Energy.

[215] Tien N.T., Jeon S., Kim D.I., Trung T.Q., Jang M., Hwang B.U., Byun K.E., Bae J., Lee E., Tok J.B. (2014). A flexible bimodal sensor array for simultaneous sensing of pressure and temperature. Adv. Mater..

[216] Trung T.Q., Ramasundaram S., Hong S.W., Lee N.-E. (2014). Flexible and transparent nanocomposite of reduced graphene oxide and P(VDF-TrFE) copolymer for high thermal responsivity in a field-effect transistor. Adv. Funct. Mater..

[217] Han X., Chen X., Tang X., Chen Y.-L., Liu J.-H., Shen Q.-D. (2016). Flexible polymer transducers for dynamic recognizing physiological signals. Adv. Funct. Mater..

[218] Ferreira A., Silva J., Sencadas V., Ribelles J.L.G., Lanceros-Méndez S. (2010). Poly[(vinylidene fluoride)-co-trifluoroethylene] membranes obtained by isothermal crystallization from solution. Macromol. Mater. Eng..

[219] Young T.-H., Lu J.-N., Lin D.-J., Chang C.-L., Chang H.-H., Cheng L.-P. (2008). Immobilization of l-lysine on dense and porous poly(vinylidene fluoride) surfaces for neuron culture. Desalination.

[220] Marques L., Holgado L.A., Simoes R.D., Pereira J.D., Floriano J.F., Mota L.S., Graeff C.F., Constantino C.J., Rodriguez-Perez M.A., Matsumoto M. (2013). Subcutaneous tissue reaction and cytotoxicity of polyvinylidene fluoride and polyvinylidene fluoride-trifluoroethylene blends associated with natural polymers. J. Biomed. Mater. Res. B Appl. Biomater..

[221] Rajabi A.H., Jaffe M., Arinzeh T.L. (2015). Piezoelectric materials for tissue regeneration: A review. Acta Biomater..

[222] Ribeiro C., Sencadas V., Correia D.M., Lanceros-Mendez S. (2015). Piezoelectric polymers as biomaterials for tissue engineering applications. Colloids Surf. B Biointerfaces.

[223] Wang J.K., Xiong G.M., Luo B., Choo C.C., Yuan S., Tan N.S., Choong C. (2016). Surface modification of PVDF using non-mammalian sources of collagen for enhancement of endothelial cell functionality. J. Mater. Sci. Mater. Med..

[224] Teow Y.H., Ahmad A.L., Lim J.K., Ooi B.S. (2012). Preparation and characterization of PVDF/TiO_2_ mixed matrix membrane via in situ colloidal precipitation method. Desalination.

[225] Kang G.-d., Cao Y.-m. (2014). Application and modification of poly(vinylidene fluoride) (PVDF) membranes—A review. J. Membr. Sci..

[226] Sheikh F.A., Beigh M.A., Qadir A.S., Qureshi S.H., Kim H. (2018). Hydrophilically modified poly(vinylidene fluoride) nanofibers incorporating cellulose acetate fabricated by colloidal electrospinning for future tissue-regeneration applications. Polym. Compos..

[227] Rajavel K., Shen S., Ke T., Lin D. (2019). Achieving high bactericidal and antibiofouling activities of 2D titanium carbide (Ti_3_C_2_T_x_ ) by delamination and intercalation. 2D Mater..

[228] Tavakolmoghadam M., Mohammadi T. (2018). Application of colloidal precipitation method using sodium polymethacrylate as dispersant for TiO_2_/PVDF membrane preparation and its antifouling properties. Polym. Eng. Sci..

[229] Shamos M.H., Lavine L.S. (1967). Piezoelectricity as a fundamental property of biological tissues. Nature.

[230] Telega J.J., Wojnar R. (2002). Piezoelectric effects in biological tissues. J. Theor. Appl. Mech..

[231] Ma Z., Kotaki M., Inai R., Ramakrishna S. (2005). Potential of nanofiber matrix as tissue-engineering scaffolds. Tissue Eng..

